# A randomized controlled trial evaluating the effects of a family-centered HIV care model on viral suppression and retention in care of HIV-positive children in Eswatini

**DOI:** 10.1371/journal.pone.0256256

**Published:** 2021-08-24

**Authors:** Kim Ashburn, Caspian Chouraya, Philisiwe Khumalo, Lydia Mpango, Nobuhle Mthethwa, Rhoderick Machekano, Laura Guay, Lynne M. Mofenson

**Affiliations:** 1 Elizabeth Glaser Pediatric AIDS Foundation (EGPAF), Washington, DC, United States of America; 2 Elizabeth Glaser Pediatric AIDS Foundation, Mbabane, Eswatini; 3 Eswatini Ministry of Health, Mbabane, Eswatini; 4 Department of Epidemiology, Milken Institute School of Public Health, The George Washington University, Washington, DC, United States of America; Prince Sattam Bin Abdulaziz University, College of Applied Medical Sciences, SAUDI ARABIA

## Abstract

**Introduction:**

A family-centered care model (FCCM) providing family-based HIV services, rather than separate adult/pediatric services, has been proposed to increase pediatric retention and treatment adherence.

**Materials and methods:**

Eight health-care facilities in the Hhohho region of Eswatini were randomized to implement FCCM (n = 4) or continue standard-of-care (SOC) separate adult/pediatric clinics (n = 4). HIV-positive children and caregivers were enrolled; caregiver interview and child/caregiver chart abstraction were done at enrollment and every three months; pediatric viral load was evaluated at enrollment and every six months through 12 months. Because of study group differences in 12-month viral load data availability (89.4% FCCM and 72.0% SOC children had 12-month viral load), we used three separate analyses to evaluate the effects of FCCM on children’s viral suppression (<1,000 copies/mL) and undetectable virus (<400 copies/mL) at 12 months. In the first analysis, all children with missing viral outcome data were excluded from the analysis (modified intent to treat, mITT). The second analysis used inverse probability of missingness weighted logistic regression to estimate the effect of FCCM on 12-month viral outcomes compared to SOC (weighted mITT). For the third approach, missing virologic outcome data were imputed as virologic failure (imputed ITT). We also examined factors associated with viral suppression at 12 months using multivariable logistic regression.

**Results:**

We enrolled 379 HIV-positive children and 363 caregivers. Among all children at enrollment, viral suppression and undetectability was 78.4% and 73.9%, respectively, improving to 90.2% and 87.3% at 12 months. In mITT and weighted mITT analyses, there was no significant difference in children’s 12-month viral suppression between FCCM and SOC groups (89.2% and 91.6%, respectively). Using imputed ITT, there was a modest increase in 12-month viral suppression in FCCM versus SOC children (79.7% and 69.8%, respectively, p = 0.051) and 12-month undetectability (78.7% and 65.7%, respectively, p = 0.015). Among the 255 children suppressed at enrollment, more FCCM versus SOC children (98.0% versus 95.3%) were suppressed at 12-months, but this was not statistically significant in mITT or weighted mITT analyses, with a marginally significant difference using imputed mITT analysis (p = 0.042). A higher proportion of children suppressed at enrollment had undetectable viral load at 12 months in FCCM versus SOC children (98.0% versus 92.5%), a statistically significant difference across analytical methods. Among the 61 children unsuppressed at enrollment, achieving suppression was higher among SOC versus FCCM children, but this difference was not statistically significant and included only 38 children; and there were no significant differences in detectable viral load at 12 months.

There were no significant differences between study groups in retention or ART adherence at 12 months for children or caregivers. Factors associated with lack of viral suppression/detectability at 12 months included lack of viral suppression at enrollment and having a younger caregiver (age <25 years).

**Conclusions:**

FCCM in Eswatini was associated with a modest increase in viral suppression/undetectability at 12-months; 12-month retention and adherence did not differ by study group for children or caregivers. High levels of suppression and retention in both groups may have limited our ability to detect a difference.

**Trial registration:**

NCT03397420; ClinicalTrials.gov.

## Introduction

In Eswatini, early adoption of universal antiretroviral therapy (ART) to all individuals living with HIV regardless of clinical stage or immune status resulted in dramatic increases in individuals receiving ART, and led to improved viral suppression [1,2]. However, in 2017, viral suppression among children aged 0–14 years on ART was 73.9%, much lower than the 91.4% among adults on ART [3].

Children receiving ART are often dependent on their caregivers to attend clinic visits, receive medication and adhere to recommended regimens. In sub-Saharan Africa, the family is the main source of care and support for children and maintaining the health and well-being of the family unit as a whole provides benefits to adults as well as children [4]. Family-centered HIV care models have emerged as an approach to deliver comprehensive care to all HIV-positive family members together in the same clinic visit, rather than providing HIV services to adults and children in separate clinics [5].

While these models are not new, few studies provide rigorous data on the role of family-centered care on pediatric HIV outcomes. Results of a 7-year retrospective analysis of data following implementation of an integrated family-focused approach to pediatric HIV care in Uganda reported a 50-fold increase in family units registered in health care, a 43-fold increase in children actively enrolled in care, and a 23-fold increase in child receiving ART [6]. In a study of women initiating ART for prevention of mother-to-child HIV transmission (PMTCT) in 12 HIV care and treatment programs in 8 sub-Saharan countries, loss to follow up was significantly greater among HIV-positive women who did not have an HIV-positive family member co-enrolled in care compared to those with a family member co-enrolled (19% versus 3 to 8% after 36 months, respectively) [7]. These limited data suggest a family-focused care approach may lead to improved retention, ART adherence and viral suppression for both children and adults. However, implementation of such a program has also been found to have challenges, including issues of HIV disclosure to children and to partners and difficulties in engaging male partners [8–10].

To improve pediatric HIV outcomes, the Eswatini Ministry of Health (MOH) initiated a pilot family-centered model (FCCM) intervention in 2016 in selected facilities in the Hhohho region. The primary aim of this study was to determine the effect of the FCCM model of HIV care on viral suppression and retention in care among HIV-positive children, comparing intervention versus control groups.

## Materials and methods

A prospective cohort of HIV-positive children aged 0–14 years, their caregivers and other HIV-positive family members were enrolled in the FAM CARE study to evaluate the effects of a FCCM program on viral suppression and retention in care at 12 months after enrollment. The study was conducted in the Hhohho region of Eswatini. This region contains four major referral centers (two hospitals and two health centers), each of which has a group of affiliated/referring clinics. For support and referrals, the two hospitals and two health centers form 4 clusters of care: Dvokolwako cluster and Emkhuzweni cluster (4 and 3 clinics affiliated with health centers, respectively) and Mbabane cluster and Pigg’s Peak cluster (12 and 10 clinics affiliated with hospitals, respectively). Within each cluster, the main hospital or health center and their largest referral clinic were selected to participate in the study. The two hospital clusters and two health center clusters were each randomized (“coin toss” selection by study staff) to either implement the FCCM intervention, where adults and children attend clinic visits together and receive HIV services as a family unit (1 hospital with its filter clinic and 1 health center with its filter clinic), or to the standard of care (SOC) control, where HIV care is provided to adults and children in separate clinics (1 hospital with its filter clinic and 1 health center with its filter clinic). Interviews with caregivers and medical chart abstraction were used to collect data on demographic characteristics, HIV and ART history, viral load, CD4 cell count, medical visit attendance, and drug pick-ups for HIV-positive children. Dried blood specimens (DBS) were obtained from HIV-positive children at enrollment, 6, and 12 months. Specimens were shipped monthly to the National Institute for Communicable Diseases (Sandringham, South Africa) for viral load testing using the Roche COBAS AmpliPrEP/TaqMan HIV-1 test.

This study received approval from the Population Council Institutional Review Board in the U.S. and the National Health Research Review Board in Eswatini.

### FCCM intervention

The FCCM is a differentiated service delivery (DSD) model aimed at providing more efficient and effective HIV services to promote better ART adherence and retention in care for families. At sites implementing FCCM, HIV-positive children were provided HIV services along with at least one other family member involved in the support of the child. A family member was defined as someone related to the child either by blood or adoption; or someone residing in the same household who was responsible for the child. Family members had to be willing to disclose their HIV status to other family members within FCCM, willing to attend clinic visits with the child and to support the child during clinic visits and at home. HIV-positive family members were invited to receive HIV care and treatment services at the same facility with the child, and HIV-negative caregivers received non-communicable disease health care services. All HIV-positive family members were to be seen together and receive their care together as a family unit, with their chronic care records kept in one family folder. ART medications were to be prepared for the family in advance of their visit and families were prioritized to be seen (or seen on special clinic “family days”) to decrease waiting time. If all family members were stable, one family member could pick up medications for the entire family. Families in FCCM were to be seen at least once every quarter for family units with stable children as defined in the Eswatini Integrated HIV Management Guidelines 2018. Families with children not meeting these criteria were to be seen monthly. For families with school-aged children, clinic visits were attempted to be scheduled around school holidays. Staff were trained in FCCM standard operating procedures (SOPs), which defined staff purpose, roles and responsibilities, and resources.

### Standard of care (SOC) control sites

Control sites continued to provide standard of care, where HIV services were delivered for adults and children in separate adult and pediatric clinics. Children were accompanied by adult caregivers to pediatric clinic visits, but adult HIV services were provided in the adult clinic during a separate clinic visit. Stable adults and stable older children (above 5 years) have ART refills every 3 months and a clinical review visit every 6 months. Stable younger children have monthly ART refills and a clinical review visit every 1–3 months depending on need for dose adjustments. Unstable adults and children have ART refills and clinic visits every month. In standard of care sites, clinical visits of adults and children were separate and could be different days; clinical visits of children were supposed to be accompanied by the adult caregiver.

In FCCM sites, adults and children would be seen for clinical visits as a family unit. The number of clinical visits for a young or unstable child could be more frequent (e.g., 1–3 months) than the required clinical visits for a stable adult and in such cases, in FCCM sites, full family clinical visits with all HIV positive family members (in addition to the caregiver adult accompanying the child to refill/clinical visits) were to be scheduled at least quarterly. In FCCM sites, it was always encouraged to see the child(ren), caregiver and other family members as a family unit during clinical care visits. In standard of care sites, visits for clinical care were separate for adults and children.

### Outcomes

Primary end points for this study were: 1) viral suppression or detectability among HIV-positive children at 12 months after study enrollment; and 2) retention in care of HIV positive children at 12 months after study enrollment. Viral suppression was defined as HIV RNA <1,000 copies/mL and undetectable viral load as HIV RNA <400 copies/mL. Viral load test results had a window of 1 month before and 2 months after a scheduled study visit. Retention in care was defined as attendance at the scheduled 12-month visit or within 3 months of the scheduled visit (as documented by clinic record abstraction). Children were classified as lost-to-follow-up (LTFU) if they had not been seen in the clinic for more than 90 days. The secondary objective was to identify individual and family factors associated with viral suppression, undetectable viral load, and retention in care.

ART adherence was assessed using several measures: caregiver report of ART interruption since last visit; health care provider assessment of adherence abstracted from the medical record (defined as good if >95% doses received; moderate if 85–95% doses received; and poor if <85% doses received); and last 2 drug refill pick-ups on time abstracted from the medical record.

### Study participant eligibility

All HIV-positive children under the age of 15 years receiving care at a study facility were eligible for enrollment in the study regardless of treatment status if there was at least one other HIV-positive family member residing in the household receiving services at that facility. HIV-positive children were excluded from the study if they were attending care at study facility only temporarily; had no other HIV-positive family members in the household receiving services at the study facility; or the child or caregiver had a significant medical condition that would preclude active study participation.

Written informed consent from the caregiver and assent from children age >12 years was obtained from all study participants prior to enrollment in the FAM CARE study.

### Data collection and statistical analysis

Data were collected through caregiver interviews and abstraction of facility, laboratory and patient records using electronic data collection forms programed onto iOS tablets with CliniOps software [11]. Caregivers and their children were enrolled into the FAM CARE study by trained study nurses from September 2017 to October 2018 and followed up through July 2019.

The study sample size was powered to detect a difference in viral suppression (HIV RNA <1,000 copies/mL) in children receiving ART in the FCCM sites compared to those receiving ART in SOC sites, with 80% power at 5% significance levels to detect at least a 10% increase in viral suppression with FCCM, assuming a suppression rate of 80% in the SOC sites, or a 15% increase in suppression if the SOC suppression rate was <75%. Assuming 10% loss-to-follow-up, this required a sample of 444 HIV-positive children from unique families (222 from FCCM sites and 222 from SOC sites). All participants were followed through 12 months, however, slower enrollment and budget constraints resulted in recruitment of 379 children rather than the 444 children in the original sample size estimation.

At enrollment trained study nurses interviewed caregivers to collect demographic, medical, and HIV-related information about the caregiver, the child and household members. Clinical laboratory data for caregivers, children and other HIV-positive family members were abstracted from clinic medical records. Study follow-up visits were every three months for children and their caregivers, scheduled to coincide with routine clinic visits to the extent possible. At each study visit, study nurses obtained interim clinical history by caregiver interview and medical chart abstraction. Data on clinic visit attendance and pharmacy drug pick-ups for children receiving ART were obtained by chart abstraction. DBS were obtained from HIV-positive children on ART for study viral load testing at enrollment and every 6 months.

Demographic characteristics at enrollment were summarized using means (standard deviations) for continuous variables and proportions for categorical variables, stratified by FCCM and SOC groups. We compared the distribution of individual characteristics at enrollment between the two study groups using chi-square tests for independence. We also compared individual characteristics at enrollment between children with and children without 12-month viral load data available to assess for potential attrition bias.

To estimate the effect of the FCCM on viral suppression/detectability, analyses were based on the intent-to-treat approach, where all enrolled children were analyzed according to randomization. Because there were differences between the two study groups in availability of 12-month viral load data (89.4% FCCM and 72.0% SOC children had 12-month viral load), we adopted three approaches. In the first approach, we included only children with 12-month virologic outcomes as assigned to study groups (modified intent-to-treat, mITT), only adjusting for differences in enrollment characteristics between study groups. In the second approach, we performed a mITT analysis with weighting to account for differential missingness in 12-month viral load data between study groups (weighted mITT). Due to differential 12-month viral outcome data availability between study groups, we used inverse probability of missingness weighting (IPMW) to adjust for potential bias. To estimate the weights, we first modeled the probability of missing 12-month viral load outcome as a function of enrollment characteristics including study group, viral load at enrollment, and child knowledge of HIV status using logistic regression (**on-line** S1 Table). We then estimated the weights by taking the inverse of the predicted probability of missing viral load. In the final approach, we used ITT analysis in which all randomized children were included, with those missing 12-month viral outcome imputed to have had virologic failure (missing = virologic failure) (imputed ITT). In all the analyses, we estimated the effect of the FCCM and associated 95% confidence intervals using logistic regression adjusting for enrollment variables that were significantly unbalanced between the study groups. We also examined the effect of the FCCM on 12-month outcomes within strata of enrollment viral load.

To identify factors associated with viral suppression, unadjusted bivariable models were used to screen for factors to include in adjusted models. Factors with p<0.2 in the bivariable analyses were included in adjusted multivariable logistic regression models of viral suppression and undetectable viremia. Stata software version 14.0 was used for data analysis.

## Results

**Fig 1:**
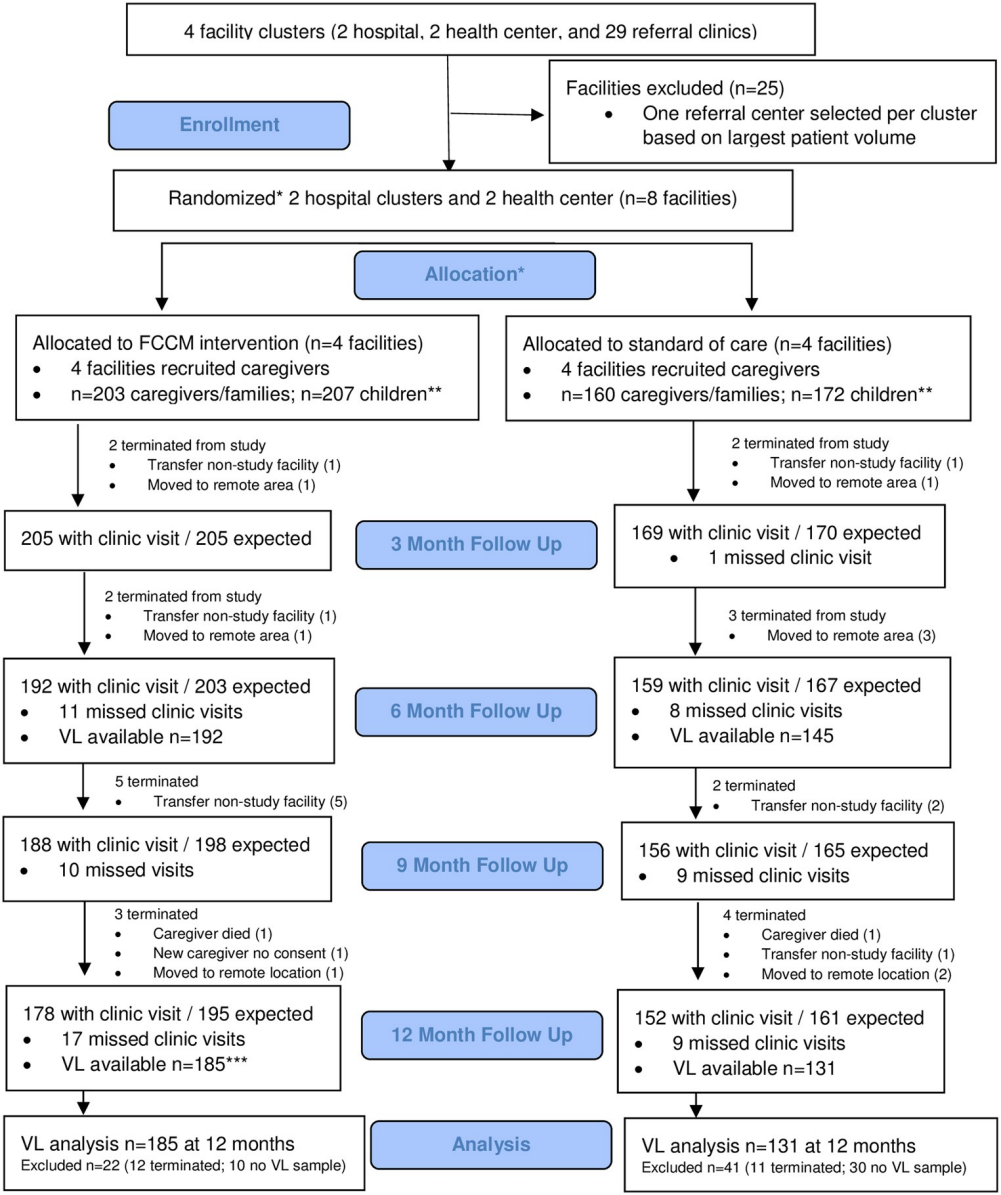
FAM CARE study consort flowchart. *Randomization was at facility cluster level: 2 clusters assigned to FCCM and 2 clusters assigned to SOC groups. **Each caregiver/family could have more than 1 child. ***Children could have viral load test drawn for study but missed a clinic visit.

A total of 379 HIV-positive children under the age of 15 years (207 at FCCM and 172 at SOC sites) from 363 unique families (203 at FCCM and 160 at SOC sites) were enrolled in the study (Fig 1). A detailed analysis of participant enrollment data has been reported elsewhere [12].

### Participant characteristics at enrollment

The median age of children at enrollment was 8.6 years; nearly half of all children (47.7%) were age 5-<10 years and one third of children were age 10–14 years (Table 1). There were few differences between children in the FCCM and SOC groups at enrollment. Significantly more children in the SOC group had missed school in the previous 3 months than in the FCCM group (33.7% versus 20.8%, p = 0.005). At enrollment, 48.3% of children in the SOC group were reported by the caregiver to know their HIV status compared to 37.2% of the children in the FCCM group (p = 0.03). Significantly more children in the SOC group were reported as having missed their HIV drugs in the last 7 days compared to the FCCM group (14.5% vs. 5.9%, p = 0.006).

**Table 1:** Demographic characteristics, HIV and treatment history of HIV-positive children.

Variable	SOC (N = 172)	FCCM (N = 207)	Total (N = 379)	p-value
	n (%)	n (%)	n (%)	
Gender				
Female	81 (47.1)	106 (51.2)	187 (49.3)	0.43
Male	91 (52.9)	101 (48.8)	192 (50.7)	
Age (years)				
0 - <5	37 (21.5)	38 (18.4)	75 (19.8)	0.63
5 - <10	82 (47.7)	97 (46.9)	179 (47.2)	
10–14	53 (30.8)	72 (34.8)	125 (33.0)	
Child in school if school-aged				
Yes	125 (94.0)	155 (91.7)	280 (92.7)	0.451
No	8 (6.0)	14 (8.3)	22 (7.3)	
Child missed school in past 3 months				
No	67 (38.9)	112 (54.1)	179 (47.2)	0.005
Yes	58 (33.7)	43 (20.8)	101 (26.6)	
Not in school	47 (27.4)	52 (25.1)	99 (26.1)	
Ever been hospitalized				
Yes	51 (29.7)	55 (26.6)	106 (28.0)	0.24
No	121 (70.3)	149 (72.0)	270 (71.2)	
Unknown	0 (0.0)	3 (1.4)	3 (0.8)	
Number of hospitalizations (n = 46 SOC; n = 50 FCCM)				
1	35 (76.1)	38 (76.0)	73 (76.0)	0.55
2	9 (19.6)	8 (16.0)	17 (17.7)	
3–5	2 (4.4)	4 (8.0)	6 (6.2)	
After entering HIV care, child continually in care				
Yes	171 (99.4)	202 (97.6)	373 (98.4)	0.33
No	1 (0.6)	4 (1.9)	5 (1.3)	
Unknown	0 (0.0)	1 (0.5)	1 (0.3)	
Child knows HIV status				
Yes	83 (48.3)	77 (37.2)	160 (42.2)	0.03
No	89 (51.7)	130 (62.8)	219 (57.8)	
Treatment regimen				
LPV/r-based	56 (35.4)	60 (31.9)	116 (33.5)	0.509
NVP-based	60 (38.0)	83 (44.2)	143 (41.3)	
EFV-based	42 (26.6)	45 (23.9)	87 (25.1)	
Missed HIV drugs in last 7 days				
Yes	24 (14.5)	12 (5.9)	36 (9.8)	0.006
No	141 (85.5)	190 (94.1)	331 (90.2)	
Enrollment viral load (copies/mL)				
<1000	130 (76.5)	164 (80.0)	294 (78.4)	0.45
≥1000	40 (23.5)	41 (20.0)	81 (21.6)	
<400	123 (71.5)	155 (74.9)	278 (73.4)	0.46
≥400	49 (28.5)	52 (25.1)	101 (26.5)	

### Antiretroviral treatment at enrollment

All children were receiving ART at enrollment except one child. Median age at HIV diagnosis was 2.1 years; at ART initiation 2.7 years; and ART duration at enrollment was 4.2 years. Children in the FCCM group were older than children in the SOC group at HIV diagnosis (2.8 versus 1.7 years, p = 0.02) and ART initiation (3.4 versus 2.0 years; p = 0.01). Of the 369 children with data on whether on first- or second-line ART at enrollment, 353 (95.7%) were on first-line ART and 16 (4.3%) on second-line ART. The majority of children, 38%, were on a zidovudine (AZT)/lamivudine (3TC)/nevirapine (NVP) regimen followed by 21% abacavir (ABC)/3TC/lopinavir/ritonavir (LPV/r), 17% ABC/3TC/efavirenz (EFV), AZT/3TC/LPV/r, 11% AZT/3TC/EFV, and 13% on other regimens. There were no significant differences in ART regimen by study group (p = 0.509). Similar proportions of children in the SOC (29.2%) and FCCM (30.0%) groups reported treatment interruptions in the year prior to enrollment.

Among the 280 (74.1%) children with data on ART adherence at enrollment, reported ART adherence in the medical chart was somewhat better in children at FCCM versus SOC sites (p = 0.02): adherence was reported as good in 85.1% of FCCM and 76.5% of SOC children, moderate in 11.2% of FCCM and 10.9% of SOC children, and poor in only 3.7% of FCCM and 12.6% of SOC children. Fewer children at FCCM versus SOC sites were reported to have missed their ART drugs at least once in the last 7 days before enrollment (5.9% versus 14.5%, p = 0.006). Of the 363 children reporting ART refill pick-ups, 88.2% of children had their last 2 ART refill pick-ups on time at enrollment, with no difference by study group (p = 0.70).

### Caregiver characteristics at enrollment

Almost all caregiver characteristics were similarly distributed between the FCCM and SOC groups at enrollment (Table 2). However, the age distribution of caregivers was marginally different between study groups, with the FCCM group having more older caregivers age >30 years compared to the SOC group (77.3% vs. 67.5%, respectively, p = 0.06). Female caregivers were significantly younger than male caregivers (median age 35 [range 15–73] years versus 40 [range 27–67] years). Nearly all of the caregivers (96.7%) were living with HIV. Caregiver households had more than one adult living with HIV in 40.8% of all households and 10% had more than one child living with HIV, with no difference between study groups.

**Table 2:** Demographic characteristics, HIV and treatment history of caregivers.

Variable	SOC (N = 160)	FCCM (N = 203)	Total	p-value
	n (%)	n (%)	n (%)	
Age (years)				
Mean (SD)	36.6 (10.9)	37.7 (10.2)	37.2 (10.5)	
18–24 years	19 (11.8)	11 (5.4)	30 (8.3)	0.06
25–29 years	33 (20.6)	35 (17.2)	68 (18.7)	
30–39 years	57 (35.6)	93 (45.8)	150 (41.3)	
≥40 years	51 (31.9)	64 (31.5)	64 (31.5)	
Gender				
Female	147 (92.5)	186 (91.6)	333 (92.0)	0.77
Male	13 (7.5)	17 (8.4)	30 (8.0)	
Education				
Never attended	20 (12.5)	31 (15.3)	51 (14.1)	0.22
Primary	67 (41.8)	68 (33.5)	135 (37.2)	
Secondary	59 (36.9)	71 (35.0)	130 (35.8)	
Beyond secondary	14 (8.8)	33 (16.2)	47 (12.9)	
Marital status				
Married	65 (40.9)	95 (46.8)	160 (44.2)	0.45
Living with partner	9 (5.7)	12 (5.9)	21 (5.8)	
Single	60 (37.5)	59 (29.1)	119 (32.8)	
Divorced/widowed/separated	26 (16.3)	37 (18.2)	63 (17.4)	
HIV status				
Positive	153 (95.6)	198 (97.5)	351 (96.7)	0.31
Negative	7 (4.4)	5 (2.5)	12 (3.3)	
Number of HIV+ adults in caregiver household including caregiver				
0	4 (2.6)	2 (1.0)	6 (1.7)	0.55
1	87 (54.4)	122 (60.1)	209 (57.6)	
2+	69 (43.1)	79 (38.9)	148 (40.8)	
Number of HIV+ children in caregiver household				
1	143 (89.4)	184 (90.6)	327 (90.1)	0.56
2	13 (8.1)	16 (7.9)	29 (8.0)	
3–4	4 (2.5)	3 (1.5)	7 (2.0)	

All caregivers living with HIV were receiving ART except one: 66.0% were receiving tenofovir disoproxil fumarate (TDF)/3TC/EFV, 18.0% AZT/3TC/NVP and 12.0% other regimens, with no significant difference between study groups. There was no significant difference at enrollment between study groups in caregivers on ART with reported good adherence (92.1% overall), last 2 drugs pick-ups on time (87.7% overall) or reported ART interruptions in the past year (19.7% overall). On chart abstraction, viral load was available for only 177/363 (48.8%) HIV-positive caregivers; most recent viral load at enrollment was <1000 copies/mL in 89.8% and <400 copies/mL in 88.1%, with no difference between study groups (P = 0.65).

### Child 12-month virologic outcomes

A total of 316 children had virologic assessment at 12 months, 131/182 (72.0%) of SOC children and 185/207 (89.4%) of FCCM children. Overall, at 12 months, 285 (90.2%) children were virally suppressed (<1,000 copies/mL) and 276 (87.3%) had undetectable virus (<400 copies/mL). Table 3 summarizes the 12-month virologic outcomes by study group, evaluating the effect of the intervention overall and stratified by viral suppression at enrollment, using the three analytic methods (mITT, weighted mITT, and imputed ITT) to account for differences in availability of 12-month viral assessment between study groups.

**Table 3:** Effect of FCCM intervention on virologic suppression and undetectability at 12 months compared to standard of care, all children and stratified by whether there is viral suppression at enrollment.

12-Month Outcome	mITT			Weighted mITT		Imputed ITT			
	SOC	FCCM	aOR^1^	IPMW aOR^2^	p	SOC	FCCM	aOR^3^	p
**12-month viral suppression (<1000 copies/ml)**									
*All Children Regardless of Enrollment Viral Suppression*									
N	131	185				172	207		
VL<1000 copies/ml	120 (91.6)	165 (89.2)	0.84 (0.37–1.90)	0.87 (0.38–1.98)	0.740	120 (69.8)	165 (79.7)	1.66 (1.00–2.76)	0.051
*Children with Enrollment VL < 1000 copies/ml (suppressed)*									
N	107	148				132	166		
VL<1000 copies/ml	102 (95.3)	145 (98.0)	2.70 (0.60–12.50)	2.78 (0.60–12.50)	0.191	102 (77.3)	145 (87.3)	92.04 (1.03–4.0)	0.042
*Children with Enrollment VL> = 1000 copies/ml (unsuppressed)*									
N	24	37				40	41		
VL<1000 copies/ml	18 (75.0)	20 (54.1)	0.42 (0.13–1.38)	0.41 (0.12–1.36)	0.145	18 (45.0)	20 (48.8)	1.15 (0.46–2.90)	0.762
**12-month viral undetectability (<400 copies/ml), stratified by status of viral suppression at enrollment**									
*All Children Regardless of Enrollment Viral Suppression*									
N	131	185				172	207		
VL<400 copies/ml	113 (86.3)	163 (88.1)	1.42 (0.71–2.85)	1.48 (0.73–2.98)	0.275	113 (65.7)	163 (78.7)	1.85 (1.13–3.04)	0.015
*Children with Enrollment VL <1000 copies/ml (suppressed)*									
N	107	148				132	166		
VL<400 copies/ml	99 (92.5)	145 (98.0)	4.76 (1.09–16.67)	4.67 (1.19–20.0)	0.027	99 (75.0)	145 (87.3)	2.27 (1.18–4.55)	0.016
*Children with Enrollment VL > = 1000 copies/ml (unsuppressed)*									
N	24	37				40	41		
VL<400 copies/ml	14 (58.3)	18 (48.6)	0.82 (0.27–2.42)	0.81 (0.27–2.41)	0.699	14 (35.0)	18 (43.9)	1.39 (0.54–3.59)	0.495

aOR: Adjusted odds ration; FCCM: Family-centered care model; IPMW: Inverse probability of missingness weighting; ITT: Intention to treat; N: Number; p: p value; SOC: Standard of care; VL: Viral load.

^1^Odds Ratio adjusted for differences in study group characteristics at enrollment.

^2^ Odds Ratio adjusted for differences in study group characteristics at enrollment and weighting for outcome missingness.

^3^Odds Ratio adjusted for differences in study group characteristics at enrollment with imputed outcomes.

In the mITT analysis, among children with a 12-month virologic outcome available, viral suppression (<1,000 copies/mL) rates were similar between groups: 89.2% of the children in the FCCM group were suppressed compared to 91.6% of the children in the SOC group. Similarly, there were no significant differences in the proportion with undetectable viral load (<400 copies/mL) between FCCM and SOC group (88.1% versus 86.3%, respectively).

The analysis of enrollment characteristics associated with missing 12-month viral load data showed that while availability of the 12-month viral load data differed by study group, there were no other significant differences in enrollment characteristics of patients with and without 12-month viral load (**on-line** S1 Table). Adjusting for differential missingness of viral load between the study groups (weighted mITT), there were no statistically significant differences in viral suppression (adjusted odds ratio (aOR) = 0.87, 95% confidence interval (CI): 0.38–1.98, p = 0.740) nor undetectable viral load (aOR = 1.48, 95% CI: 0.73–2.98, p = 0.275) at 12 months between FCCM and SOC.

Based on imputed outcome ITT analysis including all children, in which missing viral load was imputed to mean viral failure, there was a borderline trend for more children in FCCM group to be suppressed (<1,000 copies/mL) at 12-months compared to children in the SOC group (79.7% versus 69.8%, respectively, p = 0.051), and children in the FCCM group were statistically more likely to have undetectable (<400 copies/mL) viral load at 12 months compared to children in the SOC group (78.7% versus 65.7%, p = 0.015).

Among the 255 children suppressed at enrollment, 96.9% were suppressed (viral load <1,000 copies/mL) at 12 months follow-up. While the proportion of children who were suppressed at enrollment and suppressed (<1,000 copies/mL) at 12 months was higher among FCCM compared to SOC children (98.0% FCCM versus 95.3% SOC), this was not statistically significant in the mITT and weighted mITT analyses, with a marginally significant difference in the imputed ITT analysis (p = 0.042) (Table 3). Among children suppressed at enrollment at 12 months, FCCM children were significantly more likely to have undetectable 12-month viral load (<400 copies/mL) compared to SOC children (98.0% FCCM versus 92.5% SOC) in all analyses (mITT, weighted mITT, and imputed ITT).

Among the 61 children who were unsuppressed (≥1,000 copies/mL) at enrollment, 62.3% achieved viral suppression (<1,000 copies/mL) at 12 months follow-up (54.1% FCCM versus 75.0% SOC). While achieving suppression was higher among SOC compared to FCCM children, this difference was not statistically significant in any of the analytic methods (mITT, weighted mITT, and imputed ITT) and included only 38 children overall (Table 3). Similarly, among children without suppression at enrollment, there were no statistically significant differences in undetectable viral load (<400 copies/mL) at 12 months follow-up by any of the analytic methods.

### Factors associated with viral suppression

Table 4 shows the univariable and adjusted associations between 12-month viral suppression and children and caregiver characteristics. In multivariable analysis adjusting for study group, child’s age, child’s awareness of HIV status, caregiver HIV status, and number of HIV positive children in the household, virologic status at enrollment and caregiver’s age at enrollment were independently associated with viral suppression at 12 months follow up. Virally suppressed children at enrollment had a more than 18-fold increase in the odds of being suppressed at 12 months compared to children who were not suppressed at enrollment (aOR = 21.29, 95% CI: 7.95–57.04, p<0.001). Children in the care of caregivers aged 30–39 years (aOR = 5.06, 95% CI: 1.10–23.40; p = 0.038) and ≥40 years (aOR = 4.45, 95% CI: 0.94–21.01, p = 0.059) had significantly higher odds of being virally suppressed compared to those children in the care of younger caregivers under the age of 25 years.

**Table 4:** Factors associated with viral suppression at 12 months.

Characteristics	VL<1000	Unadjusted OR (95% CI)	Adjusted^a^ OR (95% CI)	p-value
Child’s age				
0-<5 years (n = 58)	49 (84.5)	1	1	
5-<10 years (n = 149)	134 (89.9)	1.64 (0.67–3.99)	0.89 (0.29–2.76)	0.840
10–15 years (n = 109)	102 (93.6)	2.68 (0.94–7.61)	0.64 (0.10–3.99)	0.634
Child’s gender				
Female (n = 153)	140 (91.5)	1		
Male (n = 163)	145 (89.0)	0.75 (0.35–1.58)		
Child knows their HIV status				
Yes (n = 138)	130 (94.2)	1	1	
No (n = 178)	155 (87.1)	0.41 (0.18–0.96)	0.31 (0.06–1.50)	0.145
Enrollment viral load				
> = 1000 copies/ml (n = 61)	38 (62.3)	1	1	
<1000 copies/ml (n = 255)	247 (96.9)	18.69 (7.80–44.78)	21.29 (7.95–57.04)	<0.001
ART regimen				
LPV/r-based (n = 103)	94 (91.3)	1		
NVP-based (n = 127)	111 (87.4)	0.66 (0.28–1.57)		
EFV-based (n = 79)	74 (93.7)	1.42 (0.46–4.41)		
Child experienced side effects from ART				
Yes (n = 19)	16 (84.2)	1		
No (n = 297)	269 (90.6)	1.80 (0.49–6.56)		
Child missed school				
No (n = 153)	13 (8.5)	1		
Yes (n = 86)	7 (8.1)	1.05 (0.40–2.74)		
Not in school (n = 77)	11 (14.3)	0.56 (0.24–1.31)		
Child missed HIV drugs				
No (n = 277)	26 (9.4)	1		
Yes (n = 29)	3 (10.3)	0.90 (0.25–3.17)		
ART adherence				
>95% (n = 191)	19 (10.0)	1		
85–95% (n = 27)	1 (3.7)	2.87 (0.37–22.4)		
<85% (n = 16)	2 (12.5)	0.77 (0.16–3.66)		
Caregiver gender				
Female (n = 291)	262 (90.3)	1		
Male (n = 25)	23 (92.0)	1.27 (0.28–5.68)		
Caregiver age				
18–24 years (n = 26)	19 (73.1)	1	1	
25–29 years (n = 54)	47 (87.0)	2.47 (0.76–8.01)	3.63 (0.75–17.62)	0.109
30–39 years (n = 128)	120 (93.8)	5.53 (1.80–17.0)	5.06 (1.10–23.40)	0.038
> = 40 years (n = 108)	99 (91.7)	4.05 (1.34–12.2)	4.45 (0.94–21.01)	0.059
Marital status				
Married (n = 140)	127 (90.7)	1		
Never married (n = 119)	106 (89.1)	0.83 (0.37–1.88)		
Divorced/Widowed (n = 57)	52 (91.2)	1.06 (0.36–3.14)		
Caregiver HIV status				
Positive (n = 303)	277 (91.4)	1	1	
Negative (n = 13)	8 (61.5)	0.15 (0.04–0.49)	0.24 (0.04–1.48)	0.124
Caregiver participates in support group				
Yes (n = 29)	27 (93.1)	1		
No (n = 287)	258 (89.9)	0.66 (0.15–2.91)		
Number of positive children in household				
1 (n = 275)	250 (90.9)	1	1	
2 (n = 30)	27 (90.0)	0.90 (0.25–3.18)	0.54 (0.10–1.48)	0.467
3+ (n = 11)	8 (72.7)	0.27 (0.07–1.07)	0.92 (0.06–12.82)	0.950

^a^Adjusted for study arm and all factors with p value <0.20 on bivariate analysis.

### Retention and adherence endpoints

There were no significant differences between FCCM and SOC groups in retention in care, interruption of ART, reported adherence, or disclosure of HIV status at 12 months (Table 5). At 12 months of follow-up, 92.7% of children expected to be seen at the 12-month visit had a documented clinic visit, with no significant difference between groups (95.2% FCCM, 95.4% SOC, p = 0.94). At the 12-month visit, ART interruption was reported by 15.5% in the FCCM and 14.5% in the SOC groups (p = 0.82); good adherence was reported by 84.3% in the FCCM and 87.2% of the SOC groups (p = 0.32); and last two drug pick-ups were reported to be on time for 87.9% of FCCM and 92.4% of SOC group children (p = 0.18). The proportion of children who were reported to not know their HIV status at enrollment but reported to have learned of their HIV status during the study was low and not statistically different between groups (6.4% FCCM and 5.1% SOC. p = 0.69).

**Table 5:** Retention, adherence, and disclosure HIV-positive children during the study.

Variable	SOC	FCCM	Total	p-value
	n (%)	n (%)	n (%)	
**12-Month Retention**				
Transferred out	8 (4.6)	10 (4.8)	18	0.94
LTFU	0	0		
Died	0	0		
Retained	164 (95.4)	197 (95.2)		
**12-Month Visit ART Adherence** ^a^				
ART Interruption since last visit (caregiver reported)	19/131 (14.5)	26/168 (15.5)	45/299 (15.1)	0.82
Good Reported Adherence (abstracted from medical record)	95/109 (87.2)	102/121 (84.3)	197/230 (85.7)	0.32
Last 2 drug pick-ups on time (abstracted from medial record)	134/145 (92.4)	153/174 (87.9)	287/319 (90.0)	0.18
**New Disclosure During Study** ^b^				
New HIV disclosure to children who did not know status at enrollment	4/79 (5.1)	8/125 (6.4)	12/204 s(5.9)	0.69

^a^ Data missing for ART interruption for 55/354 (15.5%) children (30/161 SOC, 18.6%; 25/193 FCCM, 13.0%); ART adherence 124/354 (35.0%) children (52/161 SOC, 32.3%; 72/193 intervention, 37.3%); and drug pick up for 35/319 (11.0%) children (16/161 SOC, 6.2%; 19/193 FCCM, 9.8%).

^b^ Data on child disclosure status at enrollment reported by the caregiver was available for 370/379 (97.6%) children, with 210 undisclosed; data on subsequent disclosure was available for 204/210 (97.1%) (79/83 SOC, 95.2%, and 125/127 FCCM, 98.4%).

There were no significant differences at 12 months by study group in caregiver retention as measured by missed clinic visits (overall, 6.5%), good ART adherence in HIV-positive caregivers (overall, 94.0%), or last two drug pick-ups on time in HIV-positive caregivers (overall, 91.1%). Data on caregiver viral load from clinic record abstraction after enrollment was too limited to evaluate any effect on viral load in caregivers. No adverse events were reported during the study period.

## Discussion

At 12 months after enrollment into the FAM CARE study, the rates of viral suppression and undetectable viral load were not significantly different between children enrolled in the FCCM arm compared to the SOC arm in mITT and weighted mITT-based analyses. However, in the imputed ITT analysis, where missing 12-month viral data was imputed as viral failure, children in the FCCM group had marginally significant higher odds of viral suppression and significantly higher odds of undetectable viral load at 12 months compared to children in the SOC group. Thus, viral suppression was at best modestly improved by FCCM. When stratified by the viral suppression status at enrollment, this improvement with FCCM was strongest among those children who were virally suppressed at enrollment, with viral load suppression to <1,000 copies/mL and viral undetectability to <400 copies/mL significantly higher in the FCCM than control group in the imputed mITT-based analysis Viral suppression was high in both groups at enrollment (78% overall) and increased in both groups over the course of the study, with overall viral suppression rates of 90% achieved by 12 months. It is possible that the additional attention of being enrolled in a study and having viral load monitoring every six months improved viral response in the SOC group children, limiting our ability to detect differences between groups. Additionally, the small number of children without viral suppression at enrollment limited the power to detect an effect of FCCM in this group.

Retention in care at enrollment was reported as high in both groups, with 98% overall reported to have been continuously in care, and adherence as measured by on-time ART pick-up was likewise high, 90%, in both groups. At 12 months, retention in care remained high (93% overall), as did ART adherence measured by on-time ART pick-up (90% overall), caregiver report and abstraction from medical records, with no significant differences between FCCM and SOC children. Given the high level of viral suppression, retention, and adherence (as reported by caregiver and as abstracted from medical records) at enrollment in the study overall, it would have required a significantly larger sample size to be able to detect significant differences between the FCCM program and SOC control.

There was no observed effect of FCCM on caregiver retention and adherence; similar to the data in children, rates were high (over 90% retention and adherence at 12 months). While children enrolled in the study had viral load specifically drawn for the study and assays done in a central laboratory, viral load in caregivers were extracted from medical records. Despite national recommendations for yearly viral load monitoring, only about half of caregivers had viral load test results in their medical records at enrollment; it is unknown if the test was conducted but results were not recorded or if the test was not done. These findings point to an important area for program improvement in HIV services at study clinic sites.

In addition to the high rates of viral suppression, retention, and adherence in both groups at enrollment limiting statistical power to detect significant differences at 12 months between groups, the lack of a strong effect of FCCM on viral load and retention in care for children may be related to poor implementation fidelity. According to FCCM programmatic data, only 40% of all 465 families enrolled in FCCM program at the pilot sites actually attended at least one clinic visit together as a family and only 26% of families attended four clinic visits together as a family in 12 months. Attendance at FCCM was not related to age of the child; similar proportions of families with a child <10 years of age (41.9%) versus with a child >10 years (38.1%) attended at least one clinic visit together as a family. Families enrolled in FCCM program with only two HIV-positive members (e.g., caregiver and child) were more likely to have attended at least one clinic visit as a family than those with more than two HIV-positive family members (46.7% versus 19.5%). Thus, the FAM-CARE study results related to program effects may not be generalizable to FCCM programs implemented with greater fidelity to the planned FCCM package of services.

Challenges to program implementation that were noted in qualitative interview data with FCCM participants (caregivers and health workers) are published in a separate analysis [13]. These included additional costs for clinic visits; schedule conflicts for children and adolescents; limited male participation; difficulties with disclosure and limited experience counseling families. Caregivers and health workers reported discomfort with discussing sensitive sexual and reproductive health information with family members present. Adolescents in particular, preferred to receive services through the Teen Support Club or on their own versus attend the clinic visits with family members. Some men preferred to receive HIV care services alone rather than in consultations with other family members. Similar challenges to family-centered models of care have been reported in other studies [4,8,10].

It is possible that FCCM visits took longer than standard of care individual visits, depending on the size of the family. However, in the SOC sites, while individual visits may be shorter, travel to the clinic and number of individual visits would be increased given adults and children were seen separately. Study visits were aligned with clinic visits for the child and children were not required to visit sites for additional visits specifically for the study. The number of visits for stable HIV-positive adults participating at FCCM sites may have been increased in frequency compared to SOC sites, where clinical visits were required only every 6 months. However, data on duration of time spent in the facility or number of clinic visits outside of study visits for children or family members were not collected during this study.

In a study in Uganda comparing community home-based care versus facility-based family-centered approach for children, there was no significant difference in long-term survival between models of care; retention was higher in the community-based care than family-care-based model (94.8% versus 84.7%, respectively) [14]. Irrespective of model of care, children receiving ART had significantly better retention in care and survival. All the children in the FAM-CARE study were receiving ART, with high retention at 12 months. In a cluster-randomized trial of effect of a Family Clinic Day (FCD) and family-centered appointment scheduling and health education on pediatric patient retention in Uganda, the FCD program did not improve retention but was associated with improved adherence to the clinic appointment schedule [15]. Qualitative findings suggested that FCD patients benefited from health education and increased social support. Qualitative analysis of the acceptability and feasibility of the FCCM in Eswatini revealed benefits including more open discussion and mutual support among family members about HIV resulting from health workers encouragement to disclose HIV status within the family and health workers improved ability to address needs of individuals by providing care in the context of the family [13].

Prior analysis of FAM-CARE study enrollment data demonstrated that factors associated with lack of viral suppression included treatment regimen, with children receiving NVP-based ART regimens significantly less likely to have viral suppression [12]. In the current 12-month follow-up analysis, the trend for lower suppression with NVP-based ART remains present although no longer statistically significant. These results highlight the importance of programs in Eswatini and elsewhere, to monitor treatment regimens as children age and ensure WHO guidelines for optimization of ART are implemented to improve pediatric outcomes. Given the median age of 8.6 years at enrollment, most of the children in the FAM-CARE study likely started ART based on 2010 Eswatini guidelines, when NVP was a preferred first-line ART regimen for younger children, and never had their ART regimen changed. The current process for switching children’s ART regimen is lengthy, involving at least 3 sessions of Stepped-Up Adherence Counseling (SUAC) after which a repeat viral load test is done. It can take at least 3 months or more to complete the process before switching a child’s ART regimen to second- or third-line treatment. This likely contributed to the high proportion of the 61 children who were unsuppressed at study enrollment who remained unsuppressed at the 12-month follow up visit and the small percentage (4.3%) of children receiving second-line ART.

Children aged 10–15 years had the highest rate of viral suppression at 12 months (93% versus 90% aged 5-<10 years and 85% age <5 years), although these differences were not statistically significant. Although viral suppression among older adolescents has been reported to be poor, other pediatric cohorts have reported better viral suppression among young adolescents 10–15 years than younger children (under age 5 years) and older adolescents 15–18 years [16–18]. Eswatini had an existing weekend teen HIV support group for adolescents that many of the 10–15 year old children participated in and could have contributed to improved suppression in older children. Understanding the role of teen support groups in improving viral suppression and adherence among adolescents compared to FCCM or similar family-centered HIV care models would be helpful. Future studies could assess the effectiveness of teen support groups versus family centered HIV care models or evaluate the effects of family centered HIV care models for older versus younger adolescents, on retention, adherence, and viral suppression.

Children who were reported to not know their HIV status were less likely to achieve viral suppression at 12 months after enrollment (87% versus 94% for children who know their HIV status) on bivariate analysis, although this was no longer significant on multivariate analyses. Supplemental training for health providers engaged in FCCM in how to support caregivers in disclosing the child’s HIV status could improve the proportion of children who know their HIV status. Other studies have demonstrated that adherence to treatment, viral suppression and retention in care may be linked to child’s knowledge of their HIV status [19,20]. HIV disclosure to others in the household is associated with disclosure to the child [21]. Promoting disclosure among family members in a family-centered model that includes supplemental training for health workers could lead to enhanced child disclosure as well as improved pediatric HIV outcomes. Family-based psychosocial interventions have been studied to promote HIV disclosure and support, and mental health of children infected or affected by HIV and shown in pilot studies to improve disclosure and decrease depression symptoms [22–24]. However, these interventions were separate from routine clinical care and had staff with special training; incorporation of such programs into routine clinical care would be more challenging.

The FAM-CARE data showed that adolescent/young adult caregivers may need additional support in caring for children living with HIV. Providers may need additional training in counseling and identifying the needs of younger caregivers to be able to effectively support and counsel them.

There are several limitations to the study. Given slower than expected enrollment the overall sample size was lower than planned (379 versus 444) in order to adhere to the study timeline. Viral load assessments were available for significantly more FCCM versus SOC group children at 12 months follow-up (93.4% versus 80.4%, respectively). Inverse weighting (weighted mITT) was used to adjust for potential attrition bias in models evaluating FCCM effects on viral load suppression between SOC and FCCM groups, and no significant differences between study groups were found. Although analysis with imputing missing 12-month viral load data (where missing = failure) suggested a modest effect of FCCM on viral suppression and detectability, imputing missing viral suppression outcome data as viral failure has certain limitations. For children on ART who are dependent on caregivers to get to clinic visits for routine viral testing, the assumption that missing viral load data should be considered viral failure may not be accurate. This could have introduced additional bias in our results using the imputed ITT analysis. Thus, it is unclear if the FCCM program may have modestly improved children’s viral suppression compared to the SOC group, but we are confident that FCCM did not negatively affect children’s viral suppression and based on qualitative analyses previously reported, could have had other benefits for the family and health workers implementing the program [13]. Additionally, given the high levels of viral suppression, retention, and adherence at enrollment in both FCCM and SOC children, it would have required a significantly larger sample size to be able to detect a difference in these outcomes with the FCCM program. Finally, fidelity to the implementation of the FCCM was problematic, and hence an evaluation of the program as planned and fully implemented was not possible.

## Conclusion

Children had high levels of viral suppression and undetectable viremia at enrollment, which further improved by 12 months after enrollment; we found no significant differences between FCCM intervention and SOC groups except in analyses imputing missing viral load outcome data as viral failure. In particular, in the imputed m-ITT analysis, viral suppression and viral undetectablity at 12 months were significantly higher in the FCCM group for children who were already virologically suppressed at enrollment. It is possible that the FCCM intervention further reinforced existing good practices in caregiver support of treatment in the child in children already doing well. Factors associated with viral suppression at 12 months after study entry included viral load at enrollment and caregiver’s age, with similar factors associated with undetectable viremia. A significant proportion of children were receiving suboptimal NVP-based ART at enrollment, which was associated with the lowest levels of viral suppression at enrollment and at 12 months; these data were important to direct attention to the critical need to review and optimize ART regimens in all children as they age up and more potent and less toxic regimens may become available. It is critical to actively monitor program fidelity as FCCM models are implemented, ensure sufficient staffing is provided, and to proactively address difficulties to ensure the program is being implemented as designed; specific training may be needed for health care providers, particularly in supporting HIV disclosure within families. Additional support for younger-aged caregivers in the care of HIV-positive children appears important to improve pediatric outcomes. Given the somewhat higher rates of suppression in older children, evaluation of the effects of teen support clubs on HIV pediatric outcomes could illuminate how sufficient teen support groups are in ensuring ART adherence and viral suppression among older children.

## Supporting information

S1 Checklist CONSORT 2010 checklist of information to include when reporting a randomised trial*. (PDF)

S1 Table Characteristics associated with missing 12-month viral load: Comparison of enrollment characteristics between children missing 12-month viral load outcome versus children with a 12-month viral load outcome. (DOCX)

S1 Study protocol (DOCX)

S1 Dataset (ZIP)
