# Supplementary material for: A randomized controlled trial evaluating the effects of a family-centered HIV care model on viral suppression and retention in care of HIV-positive children in Eswatini
Source: PLoS One. 2021 Aug 24;16(8):e0256256. doi: 10.1371/journal.pone.0256256 (PMC8384179; doi:10.1371/journal.pone.0256256)
Supplement: S1 Table — (DOCX) [file pone.0256256.s002.docx]

**Supplemental Table.** Characteristics associated with missing 12-month viral load: comparison of enrollment characteristics between children missing 12-month viral load outcome versus children with a 12-month viral load outcome

| Characteristic | 12-month viral outcome | | p-value | Adjusted association of characteristic with missingness | |
| --- | --- | --- | --- | --- | --- |
|  | Observed  n (%) | Missing  n (%) |  | IPWM adjusted OR (95% CI) | p-value |
| Study arm: |  |  |  |  |  |
| Control | 131 (41.5) | 51 (65.1) | 0.001 | 1 |  |
| Intervention | 185 (58.5) | 22 (34.9) |  | 0.48 (0.27 – 0.87) | 0.015 |
| Baseline viral load: |  |  |  |  |  |
| >1000 copies/mL | 61 (19.3) | 20 (31.8) | 0.028 | 1 |  |
| <1000 copies/mL | 255 (80.7) | 43 (68.2) |  | 0.67 (0.35 – 1.29) | 0.232 |
| Child age: |  |  |  |  |  |
| 0-4 years | 58 (18.4) | 17 (27.0) | 0.192 | 1 |  |
| 5-9 years | 149 (47.2) | 30 (47.6) |  | 0.86 (0.41 – 1.81) | 0.699 |
| 10-15 year | 109 (34.5) | 16 (25.4) |  | 0.79 (0.34 – 1.83) | 0.584 |
| Gender |  |  |  |  |  |
| Female | 153 (48.4) | 34 (54.0) | 0.421 | 1 |  |
| Male | 163 (51.6) | 29 (46.0) |  | 0.70 (0.37 – 1.32) | 0.267 |
| Child knows HIV status: |  |  |  |  |  |
| Yes | 138 (43.7) | 22 (34.9) | 0.199 | 1 |  |
| No | 178 (56.3) | 41 (65.1) |  | 1.06 (0.56 – 2.00) | 0.853 |
| Child had side effects from ARVs: |  |  |  |  |  |
| Yes | 19 (6.0) | 4 (6.4) | 1.000 | 1 |  |
| No | 297 (94.0) | 59 (93.6) |  | 0.64 (0.17 – 2.44) | 0.515 |
| Caregiver gender: |  |  |  |  |  |
| Female | 291 (92.1) | 55 (87.3) | 0.223 | 1 |  |
| Male | 25 (7.9) | 8 (12.7) |  | 1.91 (0.74 – 4.91) | 0.180 |
| Caregiver age: |  |  |  |  |  |
| 18-24 years | 26 (8.2) | 6 (9.5) | 0.524 | 1 |  |
| 25-30 years | 54 (17.1) | 15 (23.8) |  | 1.50 (0.48 – 4.70) | 0.488 |
| 30-40 years | 128 (40.5) | 25 (39.7) |  | 1.12 (0.39 – 3.22) | 0.840 |
| >=40 years | 108 (34.2) | 17 (27.0) |  | 0.83 (0.28 – 2.51) | 0.745 |
| Caregiver HIV status: |  |  |  |  |  |
| Positive | 303 (95.9) | 60 (95.2) | 0.736 | 1 |  |
| Negative | 13 (4.1) | 3 (4.8) |  | 1.21 (0.32 – 4.60) | 0.783 |
| Participation in teen HIV support group: |  |  |  |  |  |
| Yes | 29 (9.2) | 10 (15.9) | 0.115 | 1 |  |
| No | 287 (90.8) | 53 (84.1) |  | 0.74 (0.32 – 1.66) | 0.461 |
| Caregiver marital status: |  |  |  |  |  |
| Married/live with partner | 157 (49.7) | 34 (54.0) | 0.787 | 1 |  |
| Never married | 102 (32.3) | 20 (31.8) |  | 0.86 (0.42 – 1.74) | 0.671 |
| Divorced/separated | 18 (5.7) | 4 (6.4) |  | 1.13 (0.29 – 4.37) | 0.857 |
| Widowed | 39 (12.3) | 5 (7.9) |  | 0.33 (0.07 – 1.62) | 0.172 |
|  |  |  |  |  |  |

ARVs: antiretrovirals; IPWM: inverse probability of missingness weighting; OR: odds ratio
