# Supplementary material for: A randomized controlled trial evaluating the effects of a family-centered HIV care model on viral suppression and retention in care of HIV-positive children in Eswatini
Source: PLoS One. 2021 Aug 24;16(8):e0256256. doi: 10.1371/journal.pone.0256256 (PMC8384179; doi:10.1371/journal.pone.0256256)
Supplement: S1 Study protocol — (DOCX) [file pone.0256256.s003.docx]

Project Title: Effect of family-centered model of HIV care (FAM-CARE) on viral suppression and retention in care of HIV-positive children in Swaziland

Project Note: Please note this study is funded by Project SOAR/Population Council (PC) and by the Council’s subawardee, EGPAF. Since this study does not have a Population Council principal investigator, or a partner investigator affiliated with a primary IRB, we are requesting PC IRB to review.

Principal Investigators: Dr Caspian Chouraya

Technical Director

Swaziland- Elizabeth Glaser Paediatric AIDS Foundation

RHUS Office Park- Karl Grant St

Mbabane, Swaziland

Phone: +268-2-404-7831|Cell: +268-7-641-9892 |7-802-3831

Email: [cchouraya@pedaids.org](mailto:cchouraya@pedaids.org); [cachouraya@yahoo.com](mailto:cachouraya@yahoo.com|)

Nobuble Mthethwa

National Paediatric HIV Care and Treatment Advisor

Swaziland Ministry of Health

Phone: + 268 2404 9474, cell: +268 76187951

Email: bubu.mthethwa@gmail.com

Lynne Mofenson MD

Elizabeth Glaser Pediatric AIDS Foundation

1140 Connecticut Ave., NW, Suite 200

Washington DC

Phone: +1-202-407-7970

Email: [lmofenson@pedaids.org](mailto:lmofenson@pedaids.org)

Co-investigators: Philisiwe Khumalo

Knowledge Management & QI Manager

Swaziland- Elizabeth Glaser Paediatric AIDS Foundation

RHUS Office Park – Karl Grant Street

Mbabane, Swaziland

Phone: +268 24048081

Email: pkhumalo@pedaids.org

Lydia Mpango

Senior Clinical Advisor, JSI/AIDSFree

Swaziland- Elizabeth Glaser Paediatric AIDS Foundation

RHUS Office Park – Karl Grant Street

Mbabane, Swaziland

Phone: +268-2-404-8081/cell +268-7-676-7252

Email: [Lydia_mpango@sz.jsi.com](mailto:Lydia_mpango@sz.jsi.com)

Rhoderick Machekano, PhD, MPH

Senior Biostatistician

Elizabeth Glaser Pediatric AIDS Foundation

1140 Connecticut Ave., NW, Suite 200

Washington DC

Phone: +1-202-470-6654

Email: rmachekano@pedaids.org

Leila Katirayi, MS

Research Officer

Elizabeth Glaser Pediatric AIDS Foundation

1140 Connecticut Ave., NW, Suite 200

Washington DC

Phone: +1-202-280-1647

Email: [lkatirayi@pedaids.org](mailto:lkatirayi@pedaids.org)

Kim Ashburn

Senior Research Officer

Elizabeth Glaser Pediatric AIDS Foundation

1140 Connecticut Ave., NW, Suite 200

Washington DC

Phone: +1-202-407-7119

Email: kashburn@pedaids.org

Project location: Hhohho region, Swaziland

Proposed project dates: USAID proposal approval: November 8, 2016

Expected study end date: September 2019

Study Coordinator: Nonqaba Keatimilwe, EGPAF, Swaziland

Email: nkeatimilwe@pedaids.org

Population Council Monitor: Scott Geibel, Population Council, Washington DC

**Acronyms**

ART: Antiretroviral therapy

EGPAF: Elizabeth Glaser Pediatric AIDS Foundation

FAM-CARE: Family-centered care program

IDI: In-depth interview

LTFU: Lost to follow-up

MOH: Ministry of Health

PEPFAR: President’s Emergency Plan for AIDS Relief

PMTCT: Prevention of mother-to-child HIV transmission

SOAR: Supporting Operational AIDS Research

SOP: Standard Operating Procedure

USAID: United States Agency for International Development

WHO: World Health Organization

**Definition of Terms**

A “family unit:

For analysis purposes, a “family unit” is defined as a family in which there is at least one HIV-positive child <15 years enrolled in the study receiving ART and there is >1 family member who is also HIV-positive. In the majority of instances, a family will have only one HIV-positive child on ART, and the family “unit” equals one enrolled child plus >1 HIV-positive family member. However, if a family (in intervention or control sites) has more than one HIV-positive child on ART, all children on ART are enrolled, but for sample size purposes, this will equal one enrolled “family unit”. Data from all enrolled children will be included in the analysis with adjustment potential correlations due to family units.

Family-centered care:

The family-centered care approach is a way of caring for children and their families within health services which ensures that care is planned around the whole family, not just an individual, and in which all family members are recognized as care recipients. The approach recognizes the vital role that families play in ensuring the health and well-being of infants, children, adolescents and family members of all ages.

Viral Suppression:

Viral suppression is defined as HIV RNA level below the level of assay quantification (e.g., undetectable viral load, such as HIV RNA <50 copies/mL when the lower limit of the assay is 50 copies/mL). The proportion of patients with HIV RNA >1,000 copies/mL will also be assessed separately.

1. **SUMMARY OF PROPOSED RESEARCH**

SOAR partner Elizabeth Glaser Pediatric AIDS Foundation (EGPAF) proposes to leverage existing and planned service delivery platforms in Swaziland to evaluate a model of care to improve pediatric retention and ART adherence. In the family-centered care (FAM-CARE) program, after identification of HIV infection in a child, active HIV testing of all family members is conducted and all HIV-positive family members are seen together as a unit and receive their care together. EGPAF-Swaziland is in the process of establishing a FAM-CARE service delivery program in selected health facilities in the Hhohho region of Swaziland, and roll-out of these services will provide a platform for this study. Viral load monitoring is in process of national roll-out in Swaziland. The planned study is an implementation science study to evaluate the implementation of the FAM-CARE program in Swaziland and its effect on viral suppression and retention in children living with HIV.

The proposed study will evaluate the effect of implementing a FAM-CARE program on viral suppression and retention in children through enrollment of a prospective cohort of HIV-positive children and their caregivers at sites implementing the FAM-CARE program and control sites continuing the current standard of care. The study will be conducted in four “clusters” of facilities (2 hospitals and 2 health centers and their filter clinics) in the Hhohho region of Swaziland. Two facility “clusters” (one hospital and one health center, with their filter clinics) will be randomized to initiate the FAM-CARE program with viral load monitoring and two “clusters” (one hospital and one health center, with their filter clinics) will be control standard-of care sites. A prospective cohort of HIV-positive children and their caregivers will be followed in the FAM-CARE program sites and control sites. Each child will be followed for 18 months following enrollment. The primary objective is to evaluate the effect of the FAM-CARE program on the rates of viral suppression and retention in care, comparing rates of viral suppression and retention in children enrolled in FAM-CARE vs control sites. The study will also evaluate factors associated with viral suppression and retention (including family demographic characteristics), and conduct qualitative interviews to assess the acceptability of the FAM-CARE program by caregivers and health care providers in the intervention sites.

Since the study is comparing different program models of providing standard-of-care HIV care and treatment in facilities, there is minimal risk to participants enrolled in the study. By simply reorganizing the way services are provided, without additional costs to the program, we anticipate that the FAM-CARE program should provide more efficient and effective services that promote improved retention in care, ART initiation, adherence, and viral suppression for children and also their adult family members. If the family-centered care program is found to be effective in increasing viral suppression in children on ART, EGPAF will work with the Ministry of Health to roll-out the FAM-CARE program to more facilities in the country. Furthermore, should the program to be effective, feasible and acceptable, the program should be easily transferable to other resource-limited country settings, and contribute to the global UNAIDS/PEPFAR 90-90-90 targets by ensuring viral suppression in children on ART.

1. **BACKGROUND AND RATIONALE**

While there has been good progress in prevention of mother-to-child HIV transmission, efforts to scale up antiretroviral treatment (ART) and achieve the World Health Organization (WHO) “90-90-90” targets (90% diagnosis, 90% infected on ART; 90% on ART with viral suppression) have been less robust for children compared with adults (1,2). These discrepancies reflect substantial gaps in essential services and numerous missed opportunities to engage children in care and provide effective ART.

With the 2016 WHO recommendations for universal treatment for all HIV-positive individuals regardless of clinical or immune status, HIV-positive parents and their children will all require ART (3). HIV infection in sub-Saharan Africa occurs in the context of the family, given the predominantly heterosexual nature of HIV transmission and transmission of HIV during pregnancy, delivery and breastfeeding. The family is the basic unit of care for children, with the health and well-being of children inextricably linked to their parents’/caregivers’ physical, emotional and social health; a major aim of ART is to keep the family unit alive and well, benefitting parents and also improving the well-being of their children (4). However, most ART services engage patients as individuals, with little attention to their family or other social contexts. Provision of ART has generally entailed separate adult and pediatric clinics because different areas of expertise and service organization have been required for care of children and adults and because prior treatment guidelines were dependent on CD4 count or WHO clinical stage for ART eligibility and hence parents and children may have qualified for treatment months or years apart. However, comprehensive pediatric HIV treatment, care and support will likely not be achieved if the child is not placed in context of their family. A new paradigm has emerged, with provision of family-centered care that includes all HIV-positive family members and addresses the comprehensive health needs of all the family members, particularly the mother and child (5). Health care services that engage families may offer advantages in terms of improving retention in care and adherence with ART over the long run (6,7).

However, there is a paucity of data on the role of family-focused care on pediatric and adult treatment outcomes. A 7-year retrospective analysis of data following implementation of an integrated family-focused approach to pediatric HIV care in 10 health facilities and 10 community clinics in Uganda reported a 50-fold increase in family units registered in health care (from 70 to 3653), a 43-fold increase in children actively enrolled in care (from 86 to 3726) and a 23-fold increase in children receiving ART (from 86 to 2015) (8). In a study that examined the association between co-enrollment of HIV-positive family members into care and outcomes of women initiating ART for PMTCT in 12 HIV care and treatment programs in 8 sub-Saharan African countries, the risk of loss to follow-up was significantly greater among HIV-positive women who did not have an HIV-positive family member co-enrolled in care compared to those with a family member enrolled (19% vs 3-8% after 36 months on ART, respectively) (9). These limited data suggest that a family-focused care approach may led to improved retention, ART adherence, and viral suppression for both children and adults engaged in such care. However, implementation of such a program has also been found to have challenges, including issues of disclosure to partners and difficulties in engaging male partners (10-12).

Additionally, achieving the third “90” (90% viral suppression) is particularly challenging in children as most programs do not include pediatric viral load monitoring as part of routine care. The use of 2010/2013 WHO immunologic criteria to define treatment failure results in significant misclassification: in a study in Western Kenya, 6% (2 of 34) of children classified as treatment failure actually had undetectable viral load and 65% (45 of 69) of children thought to have treatment success had detectable viral load, supporting WHO recommendations for routine viral load monitoring (13). The potential use of viral load monitoring as a tool to promote adherence has had some evaluation among adults, but not among children. A meta-analysis of 8 studies in 8 countries found a trend for re-suppression following viral load testing with targeted adherence support for HIV-positive adults found to have detectable viremia (14). Studies are critically needed to determine how to optimally implement and use virologic testing among HIV-positive children (15).

In a recent large cross-sectional survey in Malawi and a 2013 systematic review of viral suppression in HIV-positive adults on ART, viral suppression to RNA <1,000 ranged between 61.8-71.2% (intention-to-treat analysis) (16,17). Data on virologic outcomes for children on ART in sub-Saharan Africa are limited. In a cross-sectional survey of viral suppression (RNA <80 copies/mL) among children on first-line ART for > 6 months in 10 nurse-led clinics in Lesotho, viral suppression was found in only 72.3% of 191 children; no predictors for viral outcome were identified (18). Similarly, in a study of 742 HIV-positive children initiating first-line ART in South Africa between 2008-2011, only 62.3% achieved a viral load <50 copies/mL 6 months after ART initiation (19). Somewhat better data on short-term viral response were reported among a small cohort of 69 HIV-positive children initiating ART in Zambia, with 88.5% having viral suppression (RNA <400 copies/mL) at 6 months, but this decreased to 77.8% by 24 months (20). In a descriptive study using data from a pilot program in 2012-2013 of routine viral load monitoring in 12,063 HIV-positive persons (including 580 children <10 years and 588 adolescents 10-19 years) in Shiselweni, Swaziland, 16% were found to have detectable viral load (>100 copies/mL); children had a 2.6-fold and adolescents a 3.2-fold increased odds of having a detectable viral load (29% of children <10 years and 35% of adolescents 10-19 years had detectable viremia) and were less likely to re-suppress at re-testing after adherence counseling (21). Thus, the available pediatric data suggests viral suppression below the level of assay quantification (RNA <50-400 copies/mL) is observed in 62-78% of children after >6-24 months of ART. In a pooled analysis of data from 5,485 HIV-positive children who initiated ART at 7 South African treatment programs with viral load monitoring, the probability of having a single viral load measurement >1,000 copies/mL was 16.9% at 1 year (95% CI 15.8-18.1) and 32.1% (95% CI 30.2-34.1) at 3 years after starting ART; the frequency of ART failure (two viral load >1,000 copies/mL) was 19.3% by 36 months (22). There are many factors that contribute poor overall virologic response to ART and problems with retention and ART adherence in children, many of which stem from the fact that children are dependent upon adults administration of their medications and attendance at clinic appointments; a family-focused program of care may serve to improve both pediatric retention in care and viral suppression.

EGPAF has been supporting the Kingdom of Swaziland since 2003 to prevent mother-to-child HIV transmission (PMTCT) and to provide HIV and AIDS care and treatment services to HIV-positive children and adults. Currently, EGPAF-Swaziland supports 66 health facilities in Hhohho and Shiselweni regions of Swaziland with PMTCT, HIV testing and counseling, ART and tuberculosis services. Through PEPFAR funding, EGPAF Swaziland is planning to establish family-centered service delivery program (FAM-CARE) in selected health facilities in the Hhohho region to provide more efficient and effective services to promote better ART initiation and retention in care for adults and children. The roll-out of these services will provide a platform on which to introduce and evaluate routine HIV viral load testing and monitoring in children. Two of four health care clinic clusters in the regimen will be randomized to initiate the new FAM-CARE service delivery platform, allowing a comparison of pediatric retention and viral load suppression between FAM-CARE program and control facilities.

1. **RESEARCH GOALS AND OBJECTIVES**

The overall aim of this study is to demonstrate whether a FAM-CARE approach to HIV care will improve rates of viral suppression and retention of HIV-positive children in care.

**Primary objectives:**

1. To evaluate the effect of a FAM-CARE program of HIV care on the proportion of HIV-positive children on ART with viral suppression (defined as HIV RNA copies/mL below the level of assay detection) 18 months after enrollment.

*Hypothesis: Children in the FAM-CARE program will have improved viral suppression (undetectable viral load) 18 months after enrollment compared to children in control facilities.*

1. To evaluate the effect of a FAM-CARE program of HIV care on the proportion of HIV-positive children on ART with HIV RNA >1000 copies/mL 18 months after enrollment into the study.

*Hypothesis: Children in the FAM-CARE program will have lower rates of HIV RNA >1,000 copies/mL 18 months after enrollment compared to control facilities.*

**Secondary objectives:**

1. To evaluate the effect of a FAM-CARE program of HIV care on the proportion of children with viral suppression (undetectable HIV RNA levels) and with HIV RNA >1,000 copies/mL at 6 and 12 months after enrollment into the study.

*Hypothesis: Children in the FAM-CARE program will have improved viral suppression (undetectable RNA) and lower rates of HIV RNA >1000 copies/mL at 6 and 12 months compared to children in control facilities.*

1. Determine the individual and family factors associated with viral suppression and HIV RNA >1,000 copies/mLin children 18 months after enrollment in FAM-CARE and control facilities.
2. To evaluate the effect of a FAM-CARE program of HIV care on loss to follow-up (not seen in clinical care >3 months) and ART initiation in HIV-positive children not on ART at study entry.

*Hypothesis: Children in the FAM-CARE program will have lower rates of loss to follow-up and more children initiated on ART compared to children in control facilities.*

1. Evaluate the acceptability of the FAM-CARE program to caregivers and health care providers.

*Hypothesis: The FAM-CARE program will be acceptable to caregivers and health care providers.*

1. **EXPECTED RESULTS**

The FAM-CARE approach is a way of caring for children and their families within health services which ensures that care is planned around the whole family, not just an individual, and in which all family members are recognized as care recipients. The approach recognizes the vital role that families play in ensuring the health and well-being of infants, children, adolescents, and family members of all ages. It is hypothesized that such a family-centered care program will be successful in enrolling HIV-positive children, their HIV-positive mothers and their partners into continuous follow-up through incentivizing care-seeking within a family and reducing barriers to treatment and retention. Through clinic visits involving all HIV-positive family members, health care provider workload is decreased, as they will not have to see individual members of the family on different days; this also benefits the family by decreasing the amount of times they are required to come to the facility for clinic visits or to pick up ART.

Our study will:

- Assess the effect of the FAM-CARE program on rates of viral suppression and HIV RNA level >1,000 copies/mL in HIV-positive children on ART, and factors associated with these endpoints.
- Evaluate the effect of the FAM-CARE program on missed visits, time out of care, and loss to follow-up of HIV-positive children (pre-ART and on ART), compared to the current standard of care program, and the association of these outcomes with viral suppression.
- Assess the effect of the FAM-CARE program on ART initiation among HIV-positive children not on ART at study entry.
- Assess the feasibility and acceptability of the FAM-CARE program to caregivers and health care providers.

In the Swaziland family-centered care program, no additional staff is required for the program. By simply reorganizing the way services are provided, without additional costs to the program, the program should provide more efficient and effective services that promote improved retention in care, ART initiation, adherence, and the primary endpoint of viral suppression for children and also their adult family members. Should family-centered care prove to be effective, feasible, and acceptable, the program should be easily transferable to other resource-limited country settings, and contribute to the global UNAIDS/PEPFAR 90-90-90 targets by ensuring viral suppression in children on ART.

1. **RESEARCH DESIGN AND METHODS**

**a. Overview of Study Design**

As part of implementation of the pilot FAM-CARE program in Swaziland, health care facility clusters have been randomized to initiate the pilot FAM-CARE program (two health care facility clusters) or continue to provide the current standard of HIV care with separate pediatric and adult HIV clinics (two health care facility clusters). Our study will be an evaluation of this pilot program. Our program evaluation will enroll and follow a prospective cohort of HIV-positive children from selected FAM-CARE sites and control sites to assess the effects of the FAM-CARE program on retention and viral suppression in HIV-positive children on ART. Clinical care for the children is provided through the program and not the study; the study is to evaluate the effect of the program on pediatric viral suppression and retention in care. The FAM-CARE program (described below) is being implemented under the USAID-funded AIDSFree project; no aspects of this care program will be supported through SOAR funding. The SOAR funding is solely being used to support the research evaluation of the program’s effect on pediatric viral suppression and retention.

1. **Study Relationships/Related Project**

***Description of HIV Services and EGPAF Program in Hhohho Region***

Swaziland continues to have the highest HIV prevalence in the world of 26% among individuals aged 15-49 years (23). Nurses have been trained to provide ART, allowing decentralization of ART initiation and monitoring to the local clinic level. HIV clinics are usually held daily (at high volume sites, there may be a specific day for pediatric HIV clinic or HIV antenatal care); in many sites it is the same nurse who provides adult and pediatric care.  At the hospital or health center level, there may be a specific pediatric clinic care provider, and more complex HIV cases are followed at this secondary level.  Thus, each hospital/health center has a group of affiliated/referring clinics. After ART initiation, patients are seen at 2 weeks, and then monthly for 6 months; if stable, after 6 months, adult patients are seen every 2-3 month; thus, at minimum patient visits are at least every 3 months. Pediatric patients are generally seen monthly. While Swaziland is planning to move routine viral load monitoring of patients on ART every 6 months, currently viral load monitoring has been primarily restricted to patients with suspected treatment failure.

The country is divided into four administrative regions (Manzini, Shiselweni, Hhohho and Lubombo) and there is variation in HIV prevalence across the four regions. The study will be conducted in the Hhohho region of Swaziland. The region has a population of 282,734. Approximately 53,091 people in the region are living with HIV, with 32,704 receiving ART. Hhohho has a total of 82 facilities of which 41 are public (government and mission) while the rest are privately owned (24). The health sector service delivery system is loosely organized in a four-tier system comprising of: national referral hospitals, regional hospitals, health centers, and primary health care facilities (public health units-PHUs - and clinics). Of the 41 public facilities, 1 is a referral hospital, 1 is a regional hospital, 2 are health centers and the rest are clinics and PHUs.

For easier support and referrals, the two hospitals and two health centers form 4 clusters of care facilities with their affiliated “filter” clinics in the Hhohho region: Dvokolwako cluster and Emkhuzweni cluster (clinics affiliated with health centers) and Mbabane cluster and Pigg’s Peak cluster (clinics affiliated with hospitals). EGPAF supports 38 of the 41 public facilities in Hhohho. Support from EGPAF covers the HIV testing services, prevention of mother-to-child HIV transmission (PMTCT)/maternal neonatal child health (MNCH), tuberculosis (TB), HIV care and treatment for both adults and children. EGPAF support the facilities with lay staff (HTS counselors, Expert Clients, Cough Monitors, Data Clerks); onsite mentorship; onsite and off-site trainings; quality improvement (QI) projects; cluster meetings; pilots for promising approaches; continuing medical education sessions; data quality audits; site renovations and supply of basic needs; and psychosocial support groups.

Table 1 shows the cluster, affiliated clinics, and the number of children 0-14 years newly initiated on ART during 2015 and currently on ART as of 31 December 2015, which was used as a sampling frame for selection of the study sites, based on patient volume.

**Table 1.** Clusters with Affiliated Clinics & Number of Children Aged 0-14 Years on ART, 12/2015

|  | | **Children 0-14 Years on ART 31 December 2015** | |
| --- | --- | --- | --- |
| **Facility Name** | **Facility Type** | **Initiated on ART in 2015** | **Currently on ART** |
| **Dvokolwako Cluster** | | | |
| Dvokolwako Health Centre | Health Centre | 32 | 203 |
| Balekane Nazarene Clinic | Clinic | 9 | 36 |
| Bhalekane Prison | Clinic | 0 | 0 |
| Ekuphileni Clinic | Clinic | 6 | 6 |
| Nyonyane clinic | Clinic | 2 | 5 |
| *Total* |  | *49* | *250* |
| **Mbabane Cluster** | | | |
| Mbabane Govt | Hospital | 22 | 248 |
| Ezulwini Sat. Clinic | Clinic | 6 | 11 |
| FLAS Mbabane Clinic | Clinic | 1 | 1 |
| Hhukwini Clinic | Clinic | 0 | 7 |
| Lobamba ART Clinic | Clinic | 25 | 88 |
| Mahwalala Red Cross ART Clinic | Clinic | 0 | 29 |
| Motshane ART Clinic | Clinic | 5 | 39 |
| Nkhaba clinic | Clinic | 6 | 12 |
| Salvation Army Clinic | Clinic | 5 | 5 |
| Sidwashini Correctional | Clinic | 0 | 0 |
| Sigangeni ART Clinic | Clinic | 5 | 27 |
| Siphocosini Clinic | Clinic | 7 | 20 |
| St. Mary’s Clinic | Clinic | 2 | 1 |
| *Total* |  | *84* | *488* |
| **Emkhuzweni Cluster** | | | |
| Mkhuzweni HC | Health Centre | 28 | 159 |
| Herefords Clinic | Clinic | 6 | 34 |
| Mangweni | Clinic | 17 | 64 |
| Ndwabangeni Clinic | Clinic | 5 | 32 |
| *Total* |  | *56* | *289* |
| **Pigg's Peak Cluster** | | | |
| Pigg’s Peak Hospital | Hospital | 22 | 318 |
| Bulandzeni Clinic | Clinic | 9 | 40 |
| Bulembu Clinic | Clinic | 1 | 34 |
| Horo | Clinic | 3 | 80 |
| Maguga Clinic | Clinic | 4 | 16 |
| Malandzela Clinic | Clinic | 9 | 21 |
| Mshingishingini Clinic | Clinic | 5 | 12 |
| Ndzingeni Clinic | Clinic | 2 | 17 |
| Ngowane Clinic | Clinic | 1 | 15 |
| Ntfonjeni Clinic | Clinic | 11 | 33 |
| Pigg’s Peak Nazarene Clinic | Clinic | 0 | 1 |
| *Total* |  | *67* | *587* |

***Description of FAM-CARE Service Program***

EGPAF-Swaziland is piloting a FAM-CARE program, implemented with program funding prior to initiation of the current study. Viral load monitoring is in the process of being implemented at all HIV care sites in Swaziland. For implementation of the FAM-CARE program, EGPAF-Swaziland has randomly selected one hospital and one health center and its cluster clinics (Emkhuzweni cluster and Pigg’s Peak cluster) as FAM-CARE sites and one hospital and one health center and its filter clinics (Dvokolwako cluster and Mbabane cluster) which continue their current standard of HIV care (separate pediatric and adult HIV clinics) and serve as control comparison sites. A brief description of the FAM-CARE program that has been implemented at the selected sites is provided below.

*Enrollment of patients into the FAM-CARE program*

Children receiving HIV care and treatment services at the selected FAM-CARE facilities will be the primary starting point for identifying other HIV-positive family members to be enrolled into care. The children will be recruited primarily from the HIV care and treatment departments, and in any other units within the health facility such as maternal/child health programs or PMTCT follow-up programs. After identifying a child receiving HIV treatment services, the health care worker will talk to the mother or caregiver about the FAM-CARE program within the health facility.

The adult who has brought the child to the facility will be asked the following questions:

1. If they are also HIV-positive and receiving care and treatment services. If yes, they would be invited to participate in the FAM-CARE initiative at the facility.
2. If any of their family members (spouse, children etc.) are also HIV-positive and receiving care and treatments services. If yes, an invitation letter will be issued inviting all family members to come to the health facility to participate in the FAM-CARE program at the facility if feasible. If needed, the facility health care workers will facilitate transfer out from other facilities for family members receiving HIV care at another facility. Even if the family members are not on ART but are known to be HIV-positive, they will be invited to participate in the FAM-CARE program.
3. If any of their family members (spouse, children etc.) have not tested for HIV in the past year. If yes, an invitation letter will be issued inviting all family members to come to the health facility for HIV testing. If any member is found HIV-positive, s/he would be invited to participate in the FAM-CARE program at the facility

Additionally, HIV-positive adults receiving care and treatment services will also be used as index cases to identify HIV-positive children for enrollment into the FAM-CARE program. Each family should have at least one HIV-positive child receiving HIV treatment services.

*Eligibility criteria for enrollment into FAM-CARE program*

- Presence of at least one child (up to the age of 15 years) who is HIV-positive and receiving care at the selected facility. A child will be enrolled if newly diagnosed as HIV-positive and not yet on ART (ART-naïve) or if already known to be HIV-positive but not yet initiated treatment (ART-naïve) or if already on treatment (ART-experienced).
- Family members (someone who is related to the child either by blood or adoption. These can include the mother, father, sister(s) and brother(s), cousins) receiving HIV care and treatment services and residing in the same household as the index child.
- Willingness to disclose HIV status to other family members.

It is estimated that the majority of HIV-positive children will have an HIV-positive family member, but a small percentage may be orphans cared for in a household without other HIV-positive children or adults. Additionally, a small percentage of mothers may not want to participate in the family-centered program because of disclosure issues. We estimate that this will not be more than 10-20% of HIV-positive children. These children would continue their care at the intervention facility but outside of the FAM-CARE program (e.g., routine pediatric or adult HIV clinics).

*Provision of FAM-CARE services*

All family members will be registered in the health facility as a family unit and will have their chronic care files kept in one family folder. Appointments for HIV care and treatment services will be made on the same day for the family as a unit. It is standard for HIV-positive pediatric patients on ART to be seen for monthly visits and ART pick-up. Decisions regarding ART initiation will be based on Swaziland national treatment guidelines.

*Before a scheduled family visit* to the facility, designated health care workers and expert clients (a trained, HIV-positive lay person responsible for educating, counseling, and supporting other HIV-positive clients to understand, manage, and cope with their chronic illness at the health facility level) will pull out the family folder and prepare the ART medication for the family in advance.

*Upon arrival* at the facility, the family will be directed to a designated expert client who will take them to a consultation room where they will receive the required services. Separate consultation for individual family members may be conducted if needed for sensitive topics. In clinics the family will be prioritized over individual clients in order to decrease waiting time, whilst in hospitals or health centers the families will be seen on special days that will be set aside as family-days. If none of the family members is unwell, pre-packed medication will be dispensed by the expert client. However, if a member is unwell, the health care worker will be availed to consult and manage the member. After enrollment into the FAM-CARE program, if the family members are all stable (e.g., virally suppressed on ART), only one family member can visit the health facility to collect the medication for the entire family (except for children who might need dose adjustment based on weight). Other family members will be seen at the health facility only during the stipulated dates for monitoring and as when required. Viral load testing will be conducted on all family members at the same visit (every six months).

*After leaving the facility*, family units will be reminded of their upcoming appointments through text messages three days before the appointment date. If a family unit does not return for their appointment, they will be followed up using routine client follow up processes in the supported facilities.

**c) Overview Table of Data Gathering Activities**

| **Data Gathering Activities** | Prospective Evaluation of FAM-CARE Program | Qualitative Evaluation |  |
| --- | --- | --- | --- |
|  | Randomized study of sites providing FAM-CARE HIV care compared to control standard HIV care | Semi-structured in-depth interviews |  |
| **Study population** | HIV-positive children and their caregiver  Other HIV-positive adults in the family (not required for enrollment of children) | Caregiver, health care workers |  |
| **Sample size** | 666 HIV-positive children (333 per arm) to achieve the target sample size of 444 HIV-positive children receiving ART from unique families (222 per arm) | Individual interviews with a subset of caregivers (15-25) and health care providers (15-25) in the intervention sites |  |
| **Location of activity** | 8 health care facilities (4 control, 4 intervention) in Hhohho region, Swaziland | 4 health care facilities (intervention sites) in Hhohho region, Swaziland |  |
| **Timing** | Project Month 3 (after IRB submission) | Project Month 18 |  |
| **Method** | Data abstraction from medical records; participant interview; specimen collection | Semi-structured interview led by trained research assistant |  |
| **Informed Consent document** | - Consent form for caregiver-child participation in study cohort - Assent form for children - Consent form for medical record abstraction from HIV-positive adults in the family | - Consent form for interview participation for caregivers - Consent form for interview participation for health care providers. |  |
| **Study Instrument** | Data collection tools include:   - At enrollment:   - Contact form   - Enrollment Forms (caregiver and child)   - Family Clinical Data Abstraction Form   - Child Clinical Data Abstraction Form - At each scheduled follow-up visit:   - Follow-up forms (caregiver and child)   - Family Clinical Data Abstraction Form   - Child Clinical Data Abstraction Form - As needed:   - Missed visit form   - Maternal pregnancy-transfer to MNCH care form   - Child mortality form   - Family member mortality form   - Termination form   - Study viral load specimen tracking form   - Study viral load result form | - Qualitative interview guides for caregivers - Qualitative interview guides for health care providers |  |

1. **Data Gathering Activity 1: Prospective Cohort Enrollment to Evaluate the FAM-CARE Program**
2. **Subject Population**

The Hhohho region consists of 4 clinic “clusters”; there was random selection (“coin toss” selection) of “clusters” to initiate FAM-CARE vs to continue standard of care. The FAM-CARE program is being initiated at two “clusters” of clinics that were randomly selected while the other two “clusters” will continue standard of care. The Emkhuzweni and Pigg’s Peak clusters were randomly selected to initiate FAM-CARE, while the Dvokolwako and Mbabane clusters will continue standard of care. For the **prospective** **study evaluation of the FAM-CARE program,** enrollment will take place at selected FAM-CARE and control sites, consisting of the hospital/ health center “parent” of the cluster plus the largest filter clinic for the cluster. Thus, study facilities will include 4 facilities that were randomly assigned to initiate the FAM-CARE program (one hospital plus one filter clinic and one health center and one filter clinic) (FAM-CARE site enrollment) and 4 facilities that were randomly assigned to remain standard of care (one hospital plus one filter clinic and one health center and one filter clinic) (control site enrollment) (see **Table 2** below for facilities/patient numbers). However, we may also expand to include additional filter clinics if necessary.

**Table 2:** Cluster Facilities & Number of Children on ART in Study Facilities

| **Facilities (that have been randomized to FAM-CARE program vs control sites) that will enroll children in the FAM-CARE program evaluation** | | **Children 0-14 Years on ART 31 December 2015** | |
| --- | --- | --- | --- |
| **Facility Name** | **Facility Type** | **Initiated on ART** | **Currently on ART** |
| **Dvokolwako Cluster (Control)** | | | |
| Dvokolwako Health Centre | Health Centre | 32 | 203 |
| Balekane Nazarene Clinic | Clinic | 9 | 36 |
| *Total* |  | *41* | *239* |
| **Mbabane Cluster (Control)** | | | |
| Mbabane Govt | Hospital | 22 | 248 |
| Lobamba ART Clinic | Clinic | 25 | 88 |
| *Total* |  | *47* | *336* |
| **Emkhuzweni Cluster (Intervention)** | | | |
| Mkhuzweni HC | Health Centre | 28 | 159 |
| Mangweni | Clinic | 17 | 64 |
| *Total* |  | *45* | *223* |
| **Pigg's Peak Cluster (Intervention)** | | | |
| Pigg’s Peak Hospital | Hospital | 22 | 318 |
| Horo | Clinic | 3 | 80 |
| *Total* |  | *25* | *398* |

We will enroll a prospective cohort of HIV-positive children <15 years with at least one additional HIV-positive family member in the household from FAM-CARE and control sites to evaluate the effect of the FAM-CARE program on viral suppression, HIV RNA level >1,000 copies/mL, and retention in care (See **Figure 1**). All HIV-positive children in clinical care, regardless of treatment status or enrollment in the FAM-CARE program, are eligible for enrollment into the study if there is at least one additional HIV-positive family member residing in the household. It is estimated that 85-90% of children in HIV care are currently receiving ART.

**Figure 1: Schema of Study Design for the Evaluation of FAM-CARE Program Design:**

**Four clusters of Health Care Facilities/Clinics in Hhohho Region Swaziland**

**Two hospital-based clusters with their filter clinics & two health center-based clusters with their filter clinics**

**Random selection of clusters** **(stratified by whether health center vs hospital cluster) to implement** **family-centered model of care or continue standard care**

**Randomized**

**Cluster Selection for Implementation**

| **Control Standard of HIV Care Continued**  *Dvokolwako Health Center cluster*  Four filter clinics  *Mbabane Hospital cluster*  Twelve filter clinics |
| --- |

| **Family Centered Care Model Implementation**  *Emkhuzweni Health Center cluster*  Three filter clinics  *Pigg’s Peak Hospital cluster*  Ten filter clinics |
| --- |

**Prospective study enrollment**

Will be done within the randomized hospital or health center

“parent” of the cluster plus the largest filter clinic in the cluster

| **Study Arm 1 Cohort Enrollment**  **(Intervention - Family-Center Care)**  **Emkhuzweni Health Center**  **Mangweni Clinic**  **Pigg’s Peak Hospital**  **Horo Clinic** |
| --- |

| **Study Arm 2 Cohort Enrollment**  **(Control – Standard HIV Care)**  **Dvokolwako Health Center**  **Balekane Nazarene Clinic**  **Mbabane Hospital**  **Lobamba ART Clinic** |
| --- |

**Enroll**

**HIV-positive child <15 years**

- **Receiving ART (endoints: viral suppression/HIV RNA >1,000 c/mL; pattern of retention)**
- **Not receiving ART (endoints: ART initiation; viral suppression /HIV RNA >1,000 c/mL; patterns of retention)**

**Follow-up: 18 months**

**Enroll**

**HIV-positive child <15 years**

- **Receiving ART (endoints: viral suppression/HIV RNA >1,000 c/mL; pattern of retention)**
- **Not receiving ART (endoints: ART initiation; viral suppression /HIV RNA >1,000 c/mL; patterns of retention)**

**Follow-up: 18 months**

Our primary endpoints of viral suppression and HIV RNA >1,000 copies/mL will be evaluated among HIV-positive children entering the study who are initiating or receiving ART. The sample size will be powered to detect a difference in viral suppression/HIV RNA >1,000 copies/mL in children receiving ART in the FAM-CARE sites to those receiving ART in control sites. For HIV-positive children entering the study who are not currently on ART, we will be able to compare retention in care and the proportion of children initiating ART between those enrolled in the FAM-CARE sites to those in control sites.

At FAM-CARE sites, it is estimated that 10-20% of HIV-positive children may not be enrolled in the FAM-CARE program, because of disclosure or other issues within the family. These children will still be enrolled in the prospective cohort as long as there is >1 family member who is also HIV-positive; we will be able to compare suppression/ART failure/retention rates between children in FAM-CARE sites enrolled in the FAM-CARE program and not enrolled in the FAM-CARE program, although the study will not be powered based on this comparison. We will also be able to compare overall rates of viral suppression/HIV RNA >1,000 copies/mL among all children (regardless of actual participation in FAM-CARE programs) seen at facilities with the FAM-CARE program to those in control facilities.

An individual family may have more than one HIV-positive child, and more than one HIV-positive child that is receiving treatment; based on a review of pediatric enrollment at two sites, it is estimated that ~15% of HIV-positive children come from families with more than one child receiving ART. Viral suppression rates in children from families with more than one child on treatment may be correlated, particularly in the FAM-CARE program. We will create a “family identification number” that will be used at both FAM-CARE and control sites to identify children enrolled in the study who come from the same family, which will allow controlling for potential correlation during analysis.

We will enroll all HIV-positive children with >1 family member who is also HIV-positive seen at the study facilities until we have achieved the desired sample size total of 444 HIV-positive children from unique families receiving ART at enrollment (see *Statistical Methods*). We estimate we will need to enroll approximately 666 HIV-positive children into the study (333 per arm) to achieve the target sample size of 444 HIV-positive children receiving ART from unique families (222 per arm).

***Entry Criteria for Enrollment***

*Inclusion:*

- HIV-positive child aged <15 years receiving HIV care at the study facility.
  - At least one family member residing in the household is also HIV-positive and is receiving services at the study facility.

*Exclusion:*

- HIV-positive child attending care at study facility only temporarily.
- HIV-positive child with no other HIV-positive family members in household receiving services at the study facility.
- Significant medical condition in caregiver or child that would preclude active study participation.

***Recruitment***

Eligible HIV-positive children age <15 years and their caregivers from families with at least one additional HIV-positive family member residing in the household at study sites will be identified and referred by clinic staff to the site study nurse, who will explain the study and study procedures. The study nurse will conduct the informed consent procedures with eligible caregivers and informed assent discussion with older children (> 12 years to <15 years) in a private location with a witness present in the case of an illiterate participant. The informed consent form (and informed assent form when relevant) will be read to the caregiver/child and h/she will be given the opportunity to ask questions. Written informed consent will be obtained from the mother/caregiver prior to any study procedures being conducted. Mothers under the age of 18 years will be treated as mature (emancipated) minors and able to provide informed consent for themselves and their children. After obtaining written informed consent from the caregiver, the study nurse will seek verbal informed assent from children >12 to <15 years of age. Once informed consent (and informed assent as appropriate) are obtained, participants will be enrolled in the study and assigned a participant study identification number that will include a family identification designation for all enrolled family members. Enrollment will continue in each site until the targeted number of HIV-positive children have been enrolled.

Informed consent will be sought from other HIV-positive adult (>18 years) family members at both FAM-CARE and control sites for abstraction of HIV-related clinical and laboratory data from medical records. For HIV-positive adolescents between the ages of 15 through 17 years, informed consent for data abstraction from existing medical records will be obtained from the mother/caregiver. However, this will not be required to enroll a child into the study. HIV-positive adults in the family who provide informed consent will be assigned a participant study identification number that includes a family study identification designation.

*Statistical Methodology*

*Sample size estimation*

The primary study endpoints are the proportion of children on ART with viral suppression (defined as HIV RNA below the level of assay detection) and the proportion with HIV RNA >1,000 copies/mL at 18 months. The tables below show the minimum number of children receiving ART needed to demonstrate significant (0.05) effects of the FAM-CARE program on viral suppression and HIV RNA >1,000 copies/mL compared to control facility care, assuming different viral suppression and HIV RNA >1,000 copies/mL rates in the control group.

**Table 3** (viral suppression) shows the minimum sample size per arm and overall needed to demonstrate a significant FAM-CARE program effect with 80% power at 5% significance levels, assuming different levels of viral suppression in children on ART from control facilities (ranging between 65-85% suppression) and different levels of improvement that could be observed in children in FAM-CARE program sites compared to control sites. With 398 HIV-positive children receiving ART from unique families, we will have 80% power to detect at least a 10% increase in viral suppression with FAM-CARE, assuming a viral suppression rate between 65% and 80% in control children on ART.

**Table 3.** Sample size estimates for detecting FAM-CARE effects on viral suppression rates

| % Viral Suppression in Control | Minimum Effect of FAM-CARE | % Viral Suppression in FAM-CARE Sites | Alpha | Power | Sample size/ arm | Total sample size | Adjusted for 10% LTFU |
| --- | --- | --- | --- | --- | --- | --- | --- |
| .65 | .15 | .80 | .05 | 80% | 138 | 276 | 308 |
| .65 | .20 | .85 | .05 | 80% | 73 | 146 | 164 |
| .65 | .25 | .90 | .05 | 80% | 43 | 86 | 96 |
| .70 | .15 | .85 | .05 | 80% | 121 | 242 | 270 |
| .70 | .20 | .90 | .05 | 80% | 62 | 124 | 138 |
| .75 | .15 | .90 | .05 | 80% | 100 | 200 | 224 |
| .80 | .10 | .90 | .05 | 80% | 199 | 398 | 444 |
| .85 | .05 | .90 | .05 | 80% | 686 | 1372 | 1526 |

**Table 4** (HIV RNA >1,000 copies/mL) shows the minimum number of children required to detect at least a 5%, 10%, or 15% improvement with FAM-CARE care sites compared to control sites, with 80% power at 0.05 significance level, assuming different HIV RNA >1,000 copies/mL rates in control children (ranging from 10-30%).

**Table 4:** Sample size estimates for detecting FAM-CARE effects on HIV RNA >1,000 c/mL

| % HIV RNA >1,000 c/mL in Control | Minimum Effect of FAM-CARE | % HIV RNA >1,000 c/mL in FAM-CARE Sites | Alpha | Power | Sample size/ arm | Total sample size | Adjusted for 10% LTFP |
| --- | --- | --- | --- | --- | --- | --- | --- |
| .10 | -.05 | .05 | .05 | 80% | 435 | 870 | 968 |
| .15 | -.05 | .10 | .05 | 80% | 686 | 1372 | 1526 |
| .15 | -.10 | .05 | .05 | 80% | 141 | 282 | 314 |
| .20 | -.05 | .15 | .05 | 80% | 906 | 1812 | 2014 |
| .20 | -.10 | .10 | .05 | 80% | 199 | 398 | 444 |
| .20 | -.15 | .05 | .05 | 80% | 76 | 152 | 170 |
| .25 | -.10 | .15 | .05 | 80% | 250 | 500 | 556 |
| .25 | -.15 | .10 | .05 | 80% | 100 | 200 | 244 |
| .30 | -.10 | .20 | .05 | 80% | 294 | 588 | 654 |
| .30 | -.15 | .15 | .05 | 80% | 121 | 242 | 270 |

We have adjusted the effective sample size to account for a possible 10% loss to follow up over the 18-month study period. Our estimation assumes enrolment of one HIV-positive child on ART per household and that there is no correlation of children’s viral load outcomes within facilities.

Assuming 10% loss to follow-up, we plan to enroll 444 HIV-positive children receiving ART from unique family units into the study over 12 months. With this sample size, we have power to detect a minimum of 10% increase from an estimated proportion of viral suppression of 80% in the control sites to 90% in the FAM-CARE sites or 15% increase in viral suppression with FAM-CARE if control rate of suppression is 75% or lower (Table 3 highlighted rows) and a minimum reduction in HIV RNA >1,000 copies/mL rates from 20% in the control sites to 10% in the FAM-CARE sites or from 15% in the control sites to 5% in the FAM-CARE sites (Table 4 highlighted rows).

*Study sampling procedures*

In both intervention and control facilities, all HIV-positive children under 15 years of age will be enrolled into the study until the number of target “family units” as per sample size calculation is achieved. A “family unit” is defined as a family in which at least one child <15 years enrolled in the study is receiving ART and has at least one additional family member who is HIV-positive. In the majority of instances, a family will have only one HIV-positive child on ART, and the family “unit” equals one enrolled child. However, if a family (in FAM-CARE or control sites) has more than one HIV-positive child on ART, all children on ART are enrolled, but for sample size purposes, this will equal one enrolled “family unit”. Data from all enrolled children will be included in the analysis with adjustment potential correlations due to family units (see analysis plan).

HIV-positive children initiating or receiving ART at study entry will contribute to the endpoints related to viral suppression and HIV RNA >1,000 copies/mL. HIV-positive children who are not receiving ART at study entry will contribute to endpoints related to retention in care and proportion of children not on ART who initiate ART during the study.

Assuming a 20% refusal of entry into FAM-CARE, that 15% of families will have more than one HIV-positive child enrolled in the study, and 15% of children will not be receiving ART at study entry, we estimate we will need to enroll approximately 666 HIV-positive children into the study (333 per arm) to achieve the target sample size of 444 HIV-positive children receiving ART from unique families.

**ii. Research Protocol/Methods**

1. ***Study Procedures***

After obtaining written informed consent from the caregiver and verbal informed assent from children < 12 years, eligible HIV-positive children will be enrolled in the study into the FAM-CARE or control arm depending on the facility h/she attends. An enrollment visit will be conducted, during which caregivers will be interviewed to collect demographic, medical and HIV-related information about the child. Clinical/laboratory data will be abstracted from clinic medical records. The caregiver will be interviewed regarding HIV infection status of other family members and clinical/treatment history obtained for other HIV-positive family members by interview and chart abstraction. Children will be seen for a study-specific visit at enrollment and every three months by study staff. Study visits will be scheduled to coincide with routine health services visits to the child as much as possible.

At each study visit, the study nurse will obtain interim clinical history (e.g., medical/HIV history and CD4 count and, for children on treatment, ART history, current regimen, and adherence questionnaire) by caregiver interview and abstraction of clinical and laboratory data from medical records and record on study data collection tools. Clinic visit attendance and pharmacy drug pick-up for children receiving ART will be obtained by chart abstraction. Blood will be drawn from the HIV-positive children on ART for study viral load testing at enrollment and at 6, 12, and 18 months on study. HIV-positive children not on ART at enrollment who initiate ART during study follow-up will have viral load testing at ART initiation and every six months during the study. Table 3 shows the proposed schedule of data collection.

Informed consent will be sought from other HIV-positive adult (>18 years) family members for abstraction of data on HIV-related clinical and laboratory from their medical records at enrollment and approximately every 6 months. For HIV-positive adolescents between the ages of 15 through 17 years, informed consent for data abstraction from existing medical records will be obtained from the mother/caregiver. No study visits or procedures are required for HIV-positive adult or adolescent age 15 through 17 year-old family members; only data abstraction will be performed. This consent can be obtained at enrollment or any time during the study. However, this will not be required to enroll a child into the study.

***b) Schedule of Evaluations***

**Table 5** shows the schedule of evaluations.

**Table 5.** Schedule of Evaluations

| **Data Collection** | **Forms** | **Enroll-ment** | **3 mos** | **6 mos** | **9 mos** | **12 mos** | **15 mos** | **18 mos** |
| --- | --- | --- | --- | --- | --- | --- | --- | --- |
| Informed consent caregiver-child | - Caregiver-child informed consent form | X |  |  |  |  |  |  |
| Age-appropriate assent of children > 12 years | - Verbal assent form | X |  |  |  |  |  |  |
| Informed consent for medical record abstraction from HIV-positive family members | - Family member informed consent form | X (or anytime during study) |  |  |  |  |  |  |
| Demographic data | - Enrollment Form | X |  |  |  |  |  |  |
| Family HIV history | - Enrollment Form | X |  |  |  |  |  |  |
| Treatment and viral load data for HIV-positive caregivers interview and medical record abstraction ) | - Enrollment and Follow-up Visit Form - Family Member Clinical record abstraction Form | X | X | X | X | X | X | X |
| Treatment and viral load data for HIV-positive adult family members by interview (and medical record abstraction if consent is obtained) | - Family member clinical record abstraction form | X |  | X |  | X |  | X |
| *All HIV-positive children* | | | | | | | | |
| Demographics (DOB, gender) | - Child Enrollment Form | X |  |  |  |  |  |  |
| HIV history | - Child Enrollment Form | X |  |  |  |  |  |  |
| Interim (between visits) HIV history | - Child Follow-up Form |  | X | X | X | X | X | X |
| CD4 count (program data) | - Child Clinical Record Abstraction Form | X |  | X |  | X |  | X |
| Medical visit attendance timeliness/missed visits | - Child Clinical Record Abstraction Form |  | X | X | X | X | X | X |
| *HIV-positive child receiving ART** | | | | | | | | |
| ART history | - Child Enrollment Form - Child Follow-up Form | X |  |  |  |  |  |  |
| Current ART regimen | - Child Clinical Record Abstraction Form - Child Enrollment and Follow-Up Forms | X | X | X | X | X | X | X |
| Adherence questionnaire | - Child Enrollment Form - Child Follow-up Form | X | X | X | X | X | X | X |
| Pharmacy ART pick-up | - Child Clinical Record Abstraction Form | X (if already on ART) | X | X | X | X | X | X |
| Viral load | - Blood draw | X |  | X |  | X |  | X |

*Collected any time a child is initiated on ART (on ART at entry or starts ART during study)

***c) Data Collection and Management***

1. *Data collection procedures*

Study-specific visits will be conducted at the study site by the study nurse, and be timed to coincide with routine health care visits whenever possible. Medical record abstraction will be performed by the study site nurse. Study nurses will be present in each study facility and will be responsible for the collection, maintenance of study records and data, and collection of viral load specimens. Informed consent documents, study screening/enrollment logs, and participant locator forms that include identifiable information will be completed at enrollment and stored in a locked secure location in the health facility with access limited to study staff. After all data collection is completed, the study records will be stored in central storage in EGPAF-Swaziland offices under the same strict controls.

Data will be collected and entered into electronic tablets by direct data entry into a database that will be designed specifically for this study and stored on a secure web-based server. Built-in data checks will ensure that data are within a feasible range. Any out of range values will be verified by looking through the source document. In facilities that do not have wi-fi access for immediate uploading of data, data will be stored temporarily on the tablet, and subsequently transferred to the web-based server on a routine basis. There will be controlled access to the web-based database by study staff with specific IDs and permissions depending on their study role, both for the study team in EGPAF/Swaziland and EGPAF/United States.

1. *Data collection instruments*

The study data collection instruments will be designed in English and pilot tested by the study team prior to use. Some data components in the participant interview may require translation into SiSwati to ensure accurate and consistent interpretation. The management and conduct of study visits will be the responsibility of the site study nurse. The study nurse will work closely with the study coordinator, who will be responsible for oversight of the data collection by study nurses, to ensure quality of the data collected.

Caregiver-child pair information will be collected through caregiver interviews and review of facility, laboratory and patient records. Study nurses will be trained to interview caregivers and abstract data from the facility records using electronic data capture forms. Participant-specific data collection forms will be identified with the participant’s unique study identification number. Participant names will not be included in the study database. All electronic tablets will be password protected, with access to only key study personnel.

*3. Statistical Analysis Plan*

First, we will summarize baseline characteristics of participants using means (standard deviations) or medians (IQR) for continuous variables and proportions for categorical variables, stratified by FAM-CARE/control arms. We will identify characteristics that differ significantly between the two study arms. Since participation in the FAM-CARE program is voluntary, we will assess for potential selection bias in the FAM-CARE sites by comparing characteristics of families that decline to families that accept FAM-CARE.

The primary objective of this study is to evaluate the effect of a FAM-CARE program of HIV care on the proportion of HIV-positive children on ART with viral suppression (defined as viral load below the level of assay detection) and the proportion of HIV-positive children on ART with HIV RNA >1000 copies/mL 18 months after enrollment. We will estimate the respective proportions and associated confidence intervals by study arm. Below we outline potential approaches to estimating the effect of FAM-CARE depending on comparability of the study arms:

a) If the FAM-CARE program decline rate is low (say, <10%) and baseline characteristics of children in FAM-CARE and control sites are fairly similar, we will estimate the proportion of HIV-positive children on ART with viral suppression and associated confidence intervals by study arm. Using a simple chi-square test, we will compare the two proportions at 5% significance level. The proposed chi-square test assumes outcomes from enrolled children are independent.

However, there is potential correlation of viral outcomes of children from the same family as well as children from the same facility, thereby violating the assumption of independence required for validity of the chi-square test. We propose to use multilevel generalized linear models to account for the potential intra-cluster correlation due to family unit and facility. In addition, we will adjust for potential confounding that may arise due to imbalances in children’s characteristics between study arms.

b) If the FAM-CARE program decline rate is substantial (>10%) and baseline characteristics of children differ significantly between those who accept FAM-CARE and those who decline, we propose to use the propensity score method to match and compare FAM-CARE and non- FAM-CARE participants. Using data from the FAM-CARE sites, we will estimate the probability of accepting FAM-CARE program given the baseline children and family characteristics. Using special matching algorithms, we will develop a statistical control group that will be compared with the FAM-CARE group. Regression modeling will be used to estimate the effect of the FAM-CARE group compared to the control group.

c) Finally, we will perform an intent-to-treat analysis in which all children at facilities offering the FAM-CARE program will be compared to all children at facilities without FAM-CARE program.

To answer primary aim 2, we will use the same statistical approach described for specific aim 1 above.

To answer secondary objectives 1 and 3, the analysis for the primary objective described above will be repeated using the appropriate outcomes.

To identify factors associated with viral suppression and HIV RNA >1,000 copies/mL (secondary objective 2), we propose to use multilevel logistic regression models to estimate the association between viral suppression and putative factors in each study arm. Examples of factors that might be evaluated include age and gender of child, when ART initiated and ART regimen, serious illness or hospitalization, timeliness of pharmacy drug pick-up, school attendance, family size, number of other HIV-positive individuals in family, HIV treatment and clinical status of other HIV-positive family members, disclosure of HIV status within family.

**iii. Risks and Benefits to Subjects**

There may or may not be direct benefits to study participants. However, possible benefits to the participants include close monitoring that may identify problems sooner and ensuring access to viral load testing for enrolled children.

The study poses minimal safety risks to the study participants. The services are being conducted in the framework of routine HIV care services and do not subject study participants to risks outside of what would be expected within routine services. Potential risks include pain and bruising of the child through blood sampling for viral load and the potential for unintended disclosure of HIV status. All efforts will be made to conduct interviews in private to protect participant confidentiality.

**iv. Steps to Minimize Risks**

To minimize the risk for privacy breaches, no personal identifying information will be used in the study databases and computers will be password-protected, and study staff will be trained in human subjects’ protections and be required to sign confidentiality agreements. Interviews will be conducted in private areas free from general view and out of hearing reach.

To minimize the risk of discomfort to participants, it will be made clear to them during the informed consent process that they do not have to answer any questions that they don’t want to answer and that they can also stop the interview at any time. Study staff will be thoroughly trained on interviewing techniques in general and those related to this study in particular. Pediatric blood sampling will be performed by staff trained in such procedures.

1. **Confidentiality**

Caregivers and children enrolled in the study will be identified by their unique study identifier, and data collection and forms will be linked to the patient using this unique identifier only. Children from the same household will be linked by a family identifier number at both intervention and control sites.

HIV-positive adult (>18 years) and HIV-positive adolescent aged 15 through 17 years family members will also identified by a unique study identifier with a linked family identifier number. Participation of other HIV-positive adult or adolescent age 15 through 17 year family members is not required for enrollment of the child into the study.

A master document (enrollment log) that permits linking of the enrolled individuals to their unique identification number will be maintained by the study team. This master document will be kept in a locked cabinet in a locked office with limited access. Only selected members of the study team will have access to the master document based on need. This document will not be entered into the study database. The unique study identification number will also be included on the consent forms in order to verify the consent documentation process for all participants. Consent forms will be stored separately from other data collection forms. Following study completion and all analyses, paper-based study documents for all data collection activities will be destroyed after five years.

All data will be aggregated or summarized so that no individual data will be presented as part of study findings in papers or at conferences. By virtue of their presence in the clinic and data abstraction activities from existing clinic records, study staff will view records of all women attending HIV care, including those not enrolled in the study. However, individual data from non-study participants will not be abstracted/collected for study purposes. Study staff will receive ethics training to ensure compliance with human subjects’ research requirements.

**vi. Compensation**

Study participants will not receive any compensation or incentive for participating in the study.

**vii. Informed Consent Process**

At the time of enrollment, prior to the initiation of study specific procedures, written informed consent for all study activities from enrollment through the final data collection will be obtained from all mothers/caregivers of children participating in the prospective cohort by study staff trained in protocol and informed consent procedures. Mothers under the age of 18 years will be treated as mature (emancipated) minors and able to provide consent for themselves and their children. All potential participants will be informed of the study objectives and procedures, and given the opportunity to ask questions. Potential participants will be informed that enrollment into this study is purely voluntary, and will not influence access to routine medical services. Participants will also be informed that they may choose to end their participation at any time. The informed consent document will be translated into the local language (SiSwati); participants will have the option of being consented in English or SiSwati. The study nurse will read the consent form to participants to help address varying levels of literacy. Literate participants will sign and date the consent form; illiterate participants will use a thumbprint to indicate their consent and a witness who is not the same nurse consenting the patient will observe the consent process and sign and date the consent form to certify that the participant has been read the consent on this day and voluntarily agreed to take part in the study.

Verbal, age-appropriate study assent will be obtained from older children (age > 12 to <15 years). The child will be given an explanation of the proposed research procedures in a language that is appropriate to the child's age, experience, maturity, and condition, including a discussion of any discomforts and inconveniences the child may experience if h/she agrees to participate. It will be explained that if he/she objects, participation in the research will be terminated and they will not be punished or scolded.

Informed consent, using the same written consent process described for caregivers above, will be sought from other HIV-positive adult (>18 years) family members at both FAM-CARE and control sites for abstraction of HIV-related clinical and laboratory data from medical records. However, this will not be required to enroll a child into the study.

For HIV-positive adolescents between the ages of 15 through 17 years there are no study visits or procedures; the only activity is data abstraction of limited information from their medical records (e.g., antiretroviral drugs, drug-pick up, clinic attendance, laboratory data such as CD4 cell count and viral load). Informed consent for data abstraction from existing medical records for HIV-positive adolescents age 15 through 17 years will be obtained from the mother/caregiver; however, because no study procedures or visits are required, a waiver of assent of the adolescent for data abstraction is requested.

**viii. Institutional Review Board (IRB) Review**

This protocol will be reviewed by Population Council Institutional Review Board (IRB) and the Swaziland National Health Research Review Board.

**e. Data Collection Activity 2: In-Depth Interviews with Caregivers and Health Care Workers**

To understand the feasibility and acceptability of the FAM-CARE program, a qualitative evaluation in a subset of study participants and program health care providers will be conducted.

1. **Subject Population**

***Study Population:***

The qualitative component will involve semi-structured in-depth interviews (IDIs) with two populations, caregivers and health care workers (Table 6). The study seeks to understand the feasibility and acceptability of the FAM-CARE model. Caregivers enrolled in the study who have participated in the FAM-CARE program will be interviewed to understand their experience with the FAM-CARE program, what components worked well, what were the challenges experienced and what recommendations they have for improvement. The study will also interview health care providers who have been actively involved in providing family centered care in the FAM-CARE program to learn about their experience, what components worked well, what were the challenges experienced and recommendations to improve the FAM-CARE program.

**Table 6:** Qualitative data collection overview

| **Participant Group** | **Sample Size** | **Distribution by Site Level for IDIs** | **Inclusion**  **Criteria** | **Exclusion**  **Criteria** |
| --- | --- | --- | --- | --- |
| Caregivers | 15-25 IDIs | Clinic level (2):  1-4 IDIs  Health Center (1): 5-7 IDIs  Hospital (1):  8-10 IDIs | - Joined the FAM-CARE program at least 12 months prior to data collection  - Enrolled in the FAM-CARE research study | - Did not participate in FAM-CARE program  - Participated in the tool pre-testing |
| Health Care Providers | 15-25 IDIs | Clinic level (2):  1-2 IDIs  Health Center (1): 5-8 IDIs  Hospital (1):  8-13 IDIs | -Provided care in the FAM-CARE model for a minimum of 6 months prior to data collection | - Did not provide care in the FAM-CARE program  - Participated in the tool pre-testing |

***Sample Size:***

A total of 15-25 IDIs will be conducted with caregivers and 15-25 IDIs will be conducted with health care providers for a total of 30-50 semi-structured interviews. The approximate numbers for interviews is an estimate based on feasibility and expectation for reaching saturation. The range of IDIs has been allocated by facility size according to client flow and the number of health care providers servicing the facility. This is a proposed range for the number of IDIs per facility level, the actual number of IDIs per facility level may vary but the study will not conduct more than 50 IDIs.

***Recruitment Procedures:***

Caregivers will be interviewed after they have been enrolled in the family care centered program for a minimum of 12 months. To select the study population, the study team will use computer-aided simple random selection to select participant identification numbers from the electronic study database. The study nurse will contact the selected caregiver and ask them to return on a scheduled day for the interview. Caregivers returning to the facility for the purpose of the interview will receive transport reimbursement of E50.00 (approx. 4 USD). Alternative locations for the conduct of the IDI may be arranged if returning to the facility is not feasible.

Health care providers who have provided care in the FAM-CARE program for a minimum of six months prior to data collection will be recruited. The study nurse will ask the site nurse-in-charge to identify eligible health care providers and invite them to come for an interview on a selected day. We anticipate that there will not be more health care providers than the range of IDIs selected for the different facility levels. In the case that there are more health care providers available than the range of IDIs selected for the facility level, the each health care provider will be assigned a number and selected at random by lottery. Individual folded pieces of paper containing the assigned numbers will be placed in a bag or bowl and thoroughly mixed and a member of the research team will select the numbered tags from the bag or bowl equivalent to the number of IDIs needed for that that site. The study nurse will contact the selected health care provider and ask them to meet with the interviewer on a scheduled day for the interview.

1. **Research Protocol/Methods**

***Data Collection:***

Qualitative data will be collected with in-depth interviews using a semi-structured interview guide. In-depth interviews were chosen for caregivers because the study seeks to gather information about the caregiver and child’s individual experiences receiving care. The limited number of health care providers at the intervention sites did not allow for focus group discussions, therefore in-depth interviews were selected as the data collection method for this study population.

The interview guide will gather information from the clients about their experiences enrolling in the FAM-CARE program, barriers and facilitators to remaining in the FAM-CARE program and the effects of the program on ART adherence. The health care provider tool will gather similar information about the clients in addition to gathering data about the health care provider’s experience providing services in the FAM-CARE model. All interview guides will be written in English and translated into SiSwati, although participants may choose to do the interview in English if preferred.

Research assistants (RAs) will be trained in qualitative data collection methods by EGPAF staff. During the training, all study staff will be trained on research ethics, data collection, responsibilities of the study staff, overview of the protocol and general conduct within the study.

During the data collection training the interview guides will be pre-tested. Note that the interpretative nature of qualitative research allows for flexibility with data collection tools; questions may be re-worded, omitted, moved, and added depending on the responses. One of the higher volume health facilities participating in the study will be selected to pre-test the study tools. Caregivers who are requested to participate in the pre-testing of the tools will be told that the pre-testing is just a means of practicing the data collection tools and that none of the information acquired in the piloting will be used. Those who participate in the pre-testing will not be eligible to participate in the qualitative component of study (see table 6). The RAs will not record the IDIs for the pre-testing.

The size of the site will determine the number of RAs who will be at each site. Before initiating the study, the study staff will communicate with the appropriate clinic staff to inform them about the qualitative component of the study and answer any potential questions or concerns. Upon arrival at the facility the team will introduce themselves to the head nurse (or a similar role of authority) and receive guidance on the best location at each facility to conduct the IDI. RAs will secure a place as private as possible for the IDIs, preferably an extra room not being used at the health facility.

After being introduced to the recruited study participant, the RA will read the recruitment script, obtain written informed consent and interview the study participants who voluntarily agree to participate in this component of the study. Interviews are anticipated to last approximately 1 hour. All IDIs will be conducted with one RA and one study participant. All IDIs will be audio-recorded.

Data collection is anticipated to take place over approximately 4 months.

***Analysis:***

The audio tapes of the interviews will be transcribed (and simultaneously translated into English if needed) into Microsoft Word files by the research assistants who conduct the interviews. The study team will review the transcripts and create a codebook based on the findings in the transcripts. The transcripts will be uploaded and coded in the qualitative software program MAXqda or another similar qualitative analysis software. After coding is complete, data reduction and summary tables will be generated. Data will be summarized through descriptive, text-based summaries and tables by study investigators. Textural data will be carefully read by investigators to identify recurrent patterns and themes and to draw conclusions from issues connected to study questions. Results will be analyzed by group (caregivers, health care workers) and summarized into overall findings.

***Quality Control:***

To ensure data is collected uniformly among all RAs, the SOP manual will be used throughout the training and as a reference guide during data collection. Each RA will have their own copy of the SOP manual. The SOP manual will provide detailed step by step instructions for RA responsibilities, recruiting study participants, obtaining informed consent, collecting data, data management, communication and supervisory structures and other guidance as necessary.

In addition, to ensure protocol monitoring and compliance, the study coordinator will supervise the RAs and ensure that data collection is being conducted according to the protocol and guidelines specified in the SOPs. The study coordinator will be responsible for communicating directly with the study team to report any challenges in data collection. In addition, to check the quality of the data being collected, the study coordinator will observe some IDIs to ensure the appropriate conduct and adequate probing. To ensure the quality of the transcriptions, at least 10% of the transcripts will be “verified” by the study coordinator or another member of the study team by listening to the recording and reviewing the transcript simultaneously to ensure the transcripts are accurately capturing the audio recordings.

**iii. Risks and Benefits to Subjects**

Participants will not benefit directly from participating in this component of the study, although there could be benefits resulting from improvements to the HIV care program.

While the risks of participating in the study are minimal, they may include a breach of confidentiality and possible emotional discomfort for the caregivers, similar to what is outlined in the cohort section.

1. **Steps to Minimize Risks**

In order to minimize the risk for privacy breaches, no personal identifying information will be collected from participants in the interviews, study databases and computers will be password-protected, and study staff will be trained in human subjects’ protections and required to sign confidentiality agreements. In order to minimize the risk for discomfort to participants, it will be made clear to them during the informed consent process that they do not have to answer any questions that they don’t want to answer and that they can also stop the interview at any time. Study staff will be thoroughly trained on interviewing techniques in general and those related to this study in particular.

1. **Confidentiality**

The RAs will not record names of study participants in the audio-recording or enrollment forms. During data collection, participants will be referred to by study ID numbers assigned in the training to protect the participant’s identity. Data transcripts, audio recordings, and other study files will be transported to EGPAF’s main office and kept in a locked place where only members of the research team will be able to access the files.

All data will be collected and stored in password protected computers and databases. Once downloaded onto password protected computers, the audio recorded interviews will be erased from the recorders. Electronic files will be password protected and kept on a study computer that only study and data personnel, Principal Investigators and Co-Investigators will be able to access.

1. **Compensation**

Caregivers returning to the facility for the purpose of the interview will receive transport reimbursement E50.00 (approx. 4 USD).

1. **Informed Consent Process**

Prior to beginning procedures, written informed consent will be obtained by study staff trained in protocol and ethical procedures. All potential participants will be informed of the study objectives and procedures, and given the opportunity to ask questions. Potential participants will be informed that participation in this part of the study is purely voluntary, and will not influence their employment or access to routine medical services. Participants will also be informed that they may choose to end their participation at any time. Mothers or caregivers under the age of 18 years will be treated as mature (emancipated) minors and able to provide consent.

The informed consent document will be translated into the local language of SiSwati; participants will have the option of being consented in English or SiSwati. The interviewer will read the consent form to participants to help address varying levels of literacy. Literate participants will sign and date the consent form; illiterate participants will use a thumbprint to indicate their consent and a witness who is not the same person consenting the participant will observe the consent process and sign and date the consent form to certify that the participant has been read the consent on this day and voluntarily agreed to take part in the study. The witness will observe the entire consent process.

1. **Institutional Review Board (IRB) Review**

This protocol will be reviewed by Population Council Institutional Review Board (IRB) and the Swaziland National Health Research Review Board.

1. **DATA OWNERSHIP**

The products of this research, including all intellectual property, data and other materials contributed to and developed during the study will be co-owned by the Swaziland MOH an EGPAF. The study PIs have responsibility for managing the use of the study data and specimens and ensuring that all such use is complaint with the protocol and IRB-approved study activities. Any use of data or specimens outside of the approved protocol will require approval by the PIs and review by the appropriate regulatory authorities. All investigators on this study will have access to the final locked study database according to the guidelines above. All publications from data collected in Swaziland will have Swaziland co-authorship according to the level of their contributions. Primary study records will be maintained for three years after all study-specified analyses are completed. Afterwards, the study database and database documentation will be maintained and archived for future use according to the MOH and USG research policies.

**VII. PERSONNEL AND IMPLEMENTING ORGANIZATION(S)**

EGPAF and the MOH co-principal investigators will be responsible for protocol development and other study documents, overall conduct and management and the scientific and ethical integrity of the study, analysis of data, dissemination of results, and serve as the liaison for communication and coordination with ethical review boards and other stakeholders.

The study will be led by Dr. Caspian Chouraya, Technical Director for EGPAF-Swaziland and

Dr. Nobuhle Mthethwa, National Paediatric HIV Care & Treatment Advisor, Swaziland Ministry of Health (MOH). Co-investigators include: Philisiwe Khumalo, Research and Public Health Evaluations Manager for EGPAF/Swaziland, Dr. Lydia Mpango, Senior Clinical Services Advisor for AIDSFree/Swaziland, and from EGPAF-US, Dr. Lynne Mofenson, EGPAF’s Senior Technical Advisor, Dr. Rhoderick Machekano, EGPAF’s Senior Biostatistician, Leila Katirayi, Research Officer at EGPAF-DC and Kim Ashburn, Senior Research Officer at EGPAF-DC.

Dr. Chouraya is an HIV clinician specializing in HIV care and treatment in children and adults and also a researcher with over seven years of operational research experience. He will be responsible for the overall implementation and ensuring technical soundness of the project and integration of the study within the FAM-CARE program. Dr. Mthethwa is the National Paediatric HIV Care & Treatment Advisor at the Swaziland MOH, and will represent the MOH, (which would be in charge of implementing the family-based care model nationally) in the design and implementation of the study and dissemination of results. Ms. Khumalo coordinates all EGPAF/Swaziland operations research and has over six years of operational and social research experience. She will contribute to the design, obtaining ethical approval, hiring and training of study personnel, oversight of the data collection and management and ensuring proper implementation of protocol. Dr. Mpango is a HIV clinician/researcher who will be responsible for coordinating and implementation of the family-centered approach in the health facilities. Dr. Mofenson is a world renowned expert on PMTCT and HIV treatment having directed the Maternal and Pediatric Infectious Disease Branch at the Eunice Kennedy Shriver National Institute of Child Health and Human Development at the National Institutes of Health. She will provide overall support for design and protocol development, technical soundness of the project, analysis, interpretation and manuscript writing; she will also monitor study progress to ensure that objectives will be met, mentor study investigators, and liaise with in-country PIs and other investigators. Dr. Machekano, will support the statistical-related activities of this project, including development of the statistical analysis plan, review and analysis of data and write up of study findings. Ms. Katirayi is an expert in qualitative research and data analysis, and will develop the qualitative aspects of the protocol and conduct the analyses of qualitative data. Kim Ashburn is a social and behavioral scientist with expertise in gender and HIV prevention. Dr. Ashburn will provide overall support to study implementation, study monitoring and will contribute to data analysis and manuscript writing.

**VIII. TIMELINE**

| **ACTIVITY** | **Q2**  **16** | **Q3**  **16** | **Q4**  **16** | **Q1**  **17** | **Q2**  **17** | **Q3**  **17** | **Q4**  **17** | **Q1**  **18** | **Q2**  **18** | **Q3**  **18** | **Q4**  **18** | **Q1**  **19** | **Q2**  **19** |
| --- | --- | --- | --- | --- | --- | --- | --- | --- | --- | --- | --- | --- | --- |
| Protocol and data tools development | X | X |  |  |  |  |  |  |  |  |  |  |  |
| Population Council protocol review |  | X |  |  |  |  |  |  |  |  |  |  |  |
| Protocol submission to IRBs |  |  | X |  |  |  |  |  |  |  |  |  |  |
| Intervention and Study SOP development | X | X | X |  |  |  |  |  |  |  |  |  |  |
| Study training |  |  | X |  |  |  |  |  |  |  |  |  |  |
| Study enrollment |  |  |  |  | X | X | X |  |  |  |  |  |  |
| Qualitative data collection |  |  |  |  |  |  |  |  |  | X | X |  |  |
| Study follow-up |  |  |  |  | X | X | X | X | X | X | X | X |  |
| Data cleaning, analysis |  |  |  |  |  |  |  | X |  |  |  |  | X |
| Presentation of results |  |  |  |  |  |  |  | X |  |  |  |  | X |
| Final results and dissemination |  |  |  |  |  |  |  |  |  |  |  |  | X |

**IX. References**

World Health Organization. 90-90-90: an ambitious treatment target to help ends the AIDS epidemic. Geneva, Switzerland: World health Organization, 2014 (URL: <http://www.unaids.org/en/resources/documents/2014/90-90-90>)

UNAIDS. The gap report. Geneva, Switzerland: World health Organization, 2014 (URL: <http://www.unaids.org/sites/default/files/en/media/unaids/contentassets/documents/unaidspublication/2014/UNAIDS_Gap_report_en.pdf>)

World Health Organization. Consolidated guidelines on the use of antiretroviral drugs for treating and preventing HIV infection – recommendations for a public health approach – second edition, 2016. Geneva, Switzerland: World Health Organization, 2016. (Accessed August 31, 2016, at <http://www.who.int/hiv/pub/arv/arv-2016/en/>).

Leeper SC, Montague BT, Friedman J, Flanigan TP. Lessons learned from family-centered models of treatment for children living with HIV. Current approaches and future directions. J Int AIDS Soc. 2010;13 (Suppl 2):S3.

Richter L, Beyrer C, Kippax S, Heidari S. Visioning services for children affected by HIV and AIDS through a family lens. J Int AIDS Soc. 2010;13 (Suppl 2):I1

Rochat TJ, Bland R, Coovadia H, Stein A, Newell M-L. Towards a family-centered approach to HIV treatment and care for HIV-exposed children, their mothers and their families in poorly resourced settings. Future Virol. 2011;6:687-96.

Beatancourt TS, Abrams EJ, McBain R, Fawzi MCS. Family-centered approaches to the prevention of mother-to-child transmission of HIV. J Int AIDS Soc. 2010;13 (Suppl 2):S2.

Luyirika E, Towle MS, Achan J, et al. Scaling-up paediatric HIV care with an integrated, family-centered approach: an observational case study from Uganda. PLosOne. 2013;8:e69548.

Myer L, Abrams J, Zhang Y, Duong J, El-Sadr WM, Carter RJ. Family matters: co-enrollment of family members into care is associated with improved outcomes for HIV-infected women initiating antiretroviral therapy. 2014;67 (Suppl 4):S243-9.

Towne-Gold B, Ekouevi DK, Amani-Bosse C, et al. Implementing family-focused HIV care and treatment: the first 2 years’ experience of the mother-to-child transmission-plus program in Abidigan, Cote d’Ivoire. Trop Med Int Health. 2009;14:204-12.

Van Kooten Niekerk NKM, Knies MM, Howard J, et al. The first 5 years of the family clinic for HIV at Tygerberg Hospital: family demographics, survival of children, and early impact of antiretroviral therapy. J Trop Pediatr. 2005;52:3-11.

Hosegood V, Madhavan S. Data availability on men’s involvement in families in sub-Saharan Africa to inform family-centered programmes for children affected by HIV and AIDS. J Int AIDS Soc. 2010;13 (Suppl 2):S5.

Dufort EM, DeLong AK, Mann M et al. Misclassification of antiretroviral treatment failure using WHO 2006 and 2010/2013 immunololgic criteria in HIV-infected children and adolescents in Western Kenya. J Pediatr Infect Dis Soc. 2016 Apr 29 (Epub ahead of print).

Bonner K, Mezochow A, Roberts T, Ford N, Cohn J. Viral load monitoring as a tool to reinforce adherence: a systematic review. JAIDS. 2013;64:74-8.

Roberts T, Cohn J, Bonner K, Hargreaves S. Scale-up of routine viral load testing in resource-poor settings: current and future implementation challenges. Clin Infect Dis. 2016;62:1043-8.

McMahon JH, Elliott JH, Bertagnolio S, Kubiak R, Jordan MR. Viral suppression after 12 months of antiretroviral therapy in low- and middle-income countries: a systematic review. Bulletin of the World Health Organization. 2013;91:377-85E.

Maman D, Chilima B, Masiku C, et al. Closer to 90-90-90. The cascade of care after 10 years of ART scale-up in rural Malawi: a population study. J Int AIDS Soc. 2016;19:20673.

Puga D, Cerutti B, Molisana C, et al. Still far from 90-90-90: virologic outcomes of children on antiretroviral therapy in nurse-led clinics in rural Lesotho. Pediatr Infect Dis J. 2016;35:78-80.

Pillay V, Davies MA, King S, Eley B. Short-term treatment outcomes of children starting antiretroviral therapy in the intensive care unit, general medical wards and outpatient clinics at Red Cross War Memorial Children’s Hospital, Cape Town, South Africa: a retrospective cohort study. S Afr Med J 2015;105:220-7.

Van Dijk JH, Sutcliffe CG, Munsanje B, et al. HIV-infected children in rural Zambia achieve good immunologic and virologic outcomes 2 years after initiating antiretroviral therapy. PLosOne. 2011;6:e19006.

Jobanputra K, Parker LA, Azih C, et al. Factors associated with virologic failure and suppression after enhanced adherence counseling in children, adolescents, and adults on antiretroviral therapy for HIV in Swaziland. PLosOne. 2015;10:e0116144.

Davies M-A, Moultrie H, Eley B et al. Virologic failure and second-line antiretroviral therapy in children in South Africa: the IeDEA Southern Africa Collaboration. JAIDS. 2011;56:270-8.

Central Statistical Office (CSO) [Swaziland]: Swaziland Demographic and Health Survey 2006-07. *Mbabane, Swaziland: Central Statistical Office and Macro International Inc.* 2008

Swaziland Ministry of Health. 2013 Service Availability Mapping. Mbabane: Ministry of Health; 2013
